# Extracellular Vesicles for Treatment of Bone Resorption-Related Diseases in Animal Models: Systematic Review

**DOI:** 10.1007/s00223-025-01474-7

**Published:** 2026-02-17

**Authors:** Francisco Mônico Moreira, Virgínia Amorin Fróes de Moraes, Carolina dos Santos Santinoni, Graziela Garrido Mori

**Affiliations:** 1 Medicine Course, Presidente Prudente, University of Western São Paulo (UNOESTE), Presidente Prudente, São Paulo, Brazil; 2 Dentistry Course, Federal University of Santa Catarina (UFSC), Florianópolis, Brazil

**Keywords:** Bone, Bone resorption, Extracellular vesicles, Exosomes, Treatment

## Abstract

This systematic review aimed to analyze the usefulness of EV therapy in controlling bone resorption-related diseases in animal models. The study was conducted following the PRISMA guidelines. The search was conducted until November 2025 using PubMed/MEDLINE, Scopus, Cochrane Library, and OpenGrey databases to respond to the PICO question: Would therapy with EVs be efficient for the treatment of bone resorption-related diseases in vivo? The primary and secondary outcomes were the control of bone resorption and the molecular mechanisms involved, respectively. The risk of bias was examined according to the criteria of SYRCLE's RoB tool. A total of 1031 studies were reviewed, and after applying the eligibility criteria and excluding duplicates, 38 articles were included in the results. The usefulness of EVs in controlling bone resorption was established in the majority of studies. Increased levels of osteoprotegerin (OPG) and decreased levels of the pro-inflammatory cytokines, receptor activator of nuclear factor-kB ligand (RANKL), tartrate-resistant acid phosphatase (TRAP), and osteoclasts were reported. The studies also showed enhanced levels of alkaline phosphatase (ALP), runt-related transcription factor 2 (Runx2), and osteocalcin (OCN), contributing to increased bone density. EV is a promising treatment for bone resorption-related diseases in vivo. Further studies are needed to assess safety, optimal dosing, and ideal source cells, in order to confirm the findings and support potential investigations in humans.

## Introduction

Bone remodeling, or bone turnover, is characterized by the balance between bone resorption and regeneration. This physiological process is vital for physiological activities that include adequate growth, skeletal maintenance, and proper serum calcium levels [1–3].

Osteoclastogenic cytokines, such as the ligand for receptor activating factor kB nucleus (RANKL) and macrophage colony-stimulating factor (M-CSF), participate in bone turnover [2–4]. RANKL is a cytokine that binds to the receptor activator of nuclear factor kB (RANK) present on the surface of hemopoietic cells and macrophage precursors of clastic cells [2–4]. In these same cells, there is the c-fms receptor where M-CSF binds. These cytokines, linked to their receptors, promote osteoclast differentiation [2–4].

Osteoclasts, with the aid of integrins and actin fibers, attach to the bone surface and secrete cathepsins, metalloproteinases, ions, and other products, leading to the degradation of the bone matrix and hydrolysis of the collagen-rich matrix [1–6]. Subsequently, osteoprotegerin (OPG) is secreted and binds to RANKL, modifying its form and blocking the interaction with RANK. Thus, the formation of new clastic cells is blocked, and the resorption process is interrupted [5, 6]. Bone sialoprotein (BSP) contributes to bone formation, restoring the bone tissue [5, 6], and consequently maintaining balance in the bone remodeling process. Macrophages also participate in bone remodeling by secreting pro- or anti-resorptive cytokines. Type 1 (M1) macrophages secrete interleukin 1 (IL-1) and tumor necrosis factor alpha (TNF-α), which are pro-resorptive cytokines that stimulate the production of RANKL and M-CSF. Type 2 macrophages (M2), under the action of IL-4, IL-10, or IL-13, enable osteoblasts to secrete OPG [3, 5–7].

Disorders in the bone turnover process can culminate in the onset of pathologies characterized by an increase in bone mass, such as in cases of osteopetrosis, or in situations in which the rates of bone resorption are greater than those of new formation, such as in cases of osteoporosis [8–11]. Chronic inflammatory diseases also influence disorders of the bone remodeling process, as they promote the significant production of pro-resorptive cytokines such as TNF-α, IL-1, and IL-6 [9], culminating in the interruption of the balance between the actions of osteoblasts and osteoclasts.

Current treatments for pathologies characterized by high rates of bone resorption and loss of bone mass involve the use of drugs that either inhibit osteoclastic action or increase bone deposition [10–12]. Clastic cell inhibitors, such as bisphosphonates, calcitonin, monoclonal antibodies, and estrogen, have been used in medical practice for many years [10–12]; however, these drugs negatively influence osteoblastic function and bone tissue deposition, in addition to causing gastrointestinal irritation [10, 12]. Medications that increase bone deposition, such as some hormones and strontium ranelate, can cause significant complications with long-term use; these include nausea, cramps, vascular changes, and osteosarcoma [11].

In an attempt to eliminate the side effects associated with the cited medications, different methods have been proposed and developed [9–11]. Among these, “cell-free” therapies, which use extracellular vesicles extracted from different cell types, especially stem cells, can be an alternative [13, 14].

Extracellular vesicles (EVs) have similar functions to those of the cells from which they are extracted. Song et al. [10], in 2019, detected that EVs were able to inhibit osteoclastogenesis in vitro and reduce bone resorption in vivo. Hu et al. [9], in 2021, described that exosomes, a type of extracellular vesicle, were capable of affecting the polarization of macrophages and regulating the inflammatory response via RANKL. Lee et al. [13], in 2021, verified that EVs extracted from adipose stem cells participate in the suppression of osteoclast differentiation and support the secretion of cytokines that favor bone remodeling. Cui et al. [11], in 2022, concluded that EVs can collaborate with the decrease in RANKL expression and thus control the resorptive process.

Therefore, the use of EVs is considered a promising and biocompatible therapy focused on the treatment of diseases involving bone resorption. Thinking in the aspects discussed above, one emerging question is whether therapy with EVs is superior for addressing bone resorption. Additionally, important questions arise regarding the appropriate dosage, form of administration, methods for extraction and characterization, and the specific cell types from which the EVs originate. The investigation of molecular mechanisms and cell types involved in the control of bone resorption resulting from the use of EVs is also questioned. These data will be crucial for guiding future clinical practices in humans.

Thus, the objectives of the present study were, by a systematic review, to analyze the usefulness of EV therapy in controlling bone resorption-related diseases, and to recognize the molecular mechanisms participating in this process. The description of origin cells, dosage, form of administration for the use of EVs, as well as the method of extraction and characterization of them, were included in the objectives.

## Material and Methods

The systematic review was conducted following the recommendations of “The Preferred Reporting Items for Systematic Reviews and Meta-Analyses (PRISMA) [15]. This study was registered in Prospective Register of Systematic Reviews (PROSPERO CRD42024493291).

### Eligibility Criteria and PICO Question

The inclusion criteria were: (1) in vivo animal models with stimulated bone resorption, (2) studies analyzing the EVs and bone resorption, (3) comparing studies to use of EVs or not, (4) studies explaining the molecular mechanisms involved in the control of bone resorption, (5) studies detailing the dosage, form of use, and origin-cell of EVs, as well as method of extraction and characterization of EVs.

The exclusion criteria were: (1) in vivo animal models without stimulated bone resorption, (2) studies that have not analyzed bone resorption, (3) studies with the absence of a comparator control group, (4) studies that have not analyzed the molecular mechanisms involved in the resorptive process, (5) studies have not analyzed the origin cell, and method of extraction and characterization of EVs, (6) studies have not analyzed the dosage and form administration of EVs, (7) studies with EVs from tumoral cells or plants, or mimetics extracellular vesicles, (8) review articles, non-comparative studies, commentaries, editorials, case reports, case series, conference abstracts, clinical cases, and *sillico* studies. For studies with missing data, including origin cell, method of extraction and characterization of EVs, dosage, or form of administration, authors were contacted via e-mail for three times; the lack of requested information resulted in the exclusion of the article.

The specific clinical question (PICO: Population, Intervention, Comparison, Outcome) was: Would therapy with EVs be useful for the treatment of bone resorption-related diseases in vivo? In this approach, (P) represented animals with stimulated bone resorption, (I) referred to therapy with EVs, in comparison to (C) not using them, and (O) related to the control of bone resorption. The control of bone resorption and its molecular mechanism were considered the primary and secondary outcomes, respectively. The dosage, form of administration, origin cell, and method for extraction and characterization of EVs were also described.

For each study, the comparison group was considered a criterion for inclusion, and the absence of a comparative control group was considered an exclusion criterion. Control groups consisted of animals not receiving EVs treatment, such as sham-operated groups, vehicle-treated animals (PBS or other non-EV treatments that did not affect outcomes), or untreated disease models. Our analysis focuses on comparing the use and non-use of EVs; therefore, studies lacking a comparison group were excluded.

### Search Strategy and Selection Process

An electronic search was performed in the PubMed/MEDLINE, Scopus, Cochrane Library, and Open Gray Databases for the period up to November 2025. Two independent researchers (FMM and VAFM) led the search in the databases cited data, considering the following keywords: (a) exosome, (b) extracellular vesicles, and (c) bone resorption. The search strategy was based on the following crossings: #1 (exosome and bone resorption), #2 (extracellular vesicles and bone resorption), and #3 (exosome and extracellular vesicles and bone resorption). The crossing #exosome and extracellular vesicles was not executed because it included non-specific results directly related to the objectives of this study. No filters were used during searches in PubMed/MEDLINE, Cochrane Library, and Open Gray databases. For the Scopus database, the filter titles, summary, and keywords. The studies were selected by analyzing their title and abstracts regarding the inclusion and exclusion criteria. Disagreements were resolved through online or in-person dialogue, re-reading the article title and abstract, discussing inclusion or exclusion points, and reaching consensus with a third researcher (GGM). At the end of the selection of scientific articles, the level of agreement between the researchers was calculated using the Kappa statistical test [16].

### Data Collection Process and Data List

The selected studies were completely considered, and a specific analysis was executed to extract significant data related to the PICO question and the objectives of the present study. A researcher (FMM) collected the pertinent information, adding it to a table that included animal species, diseases or conditions associated with bone resorption, particulars concerning EVs (type or size, method of extraction and characterization, origin cell, dosage, and form of administration of EVs), experimental groups, period, and types of analysis, and outcomes. A standardized form containing the previous items was used for notes and data collection. Afterward, a second researcher (GGM) reviewed the information collected by reading the articles and confirming each piece of information extracted by the first author, comparing the data presented in the table with the written article. Any disagreements were resolved through dialogue, either online or in person, by re-reading the article, selecting specific data, and comparing it with previously extracted data, with a consensus reached with another researcher (VAFM).

### Data Synthesis Process

A meta-analysis was not conducted due to insufficient data. The required data were presented in graphical or image-based formats in the majority of studies, which prevented accurate extraction of means and standard deviations for the control and treatment groups. It should be noted the numerical data were available in two studies related to periodontitis. Moreover, the authors of the relevant studies were contacted via e-mail on three separate occasions; however, only three provided the requested information, two specific to osteoporosis and one to periodontitis. In addition, the parameters used in these studies did not allow for comparison among obtained data (Table).

**Table 1:** Articles excluded after full-text analysis

Author/Year	Justificative
PI et al. [58]	This study did not provide data regarding the dose, despite the author being requested to provide it via email
Li et al. [59]	This study did not provide data regarding the dose, despite the author being requested to provide it via email
Zhou et al. [60]	Study i*n vitro,* only
Cai et al. [61]	The use of Evs was evaluated only in vitro
Choi et al. [62]	Study i*n vitro,* only
Duan et al. [63]	The study evaluated the composition of vesicles extracted from sham and OVX rats and their action on cells in different conditions
Li et al. [64]	This study did not provide data regarding the dose, despite the author being requested to provide it via email
Li et al. [65]	Although authors studied osteolysis, its occurrence was due to the placement of prostheses and not a disease that induces bone resorption through its intrinsic physiopathology
Pan et al. [66]	Although authors studied osteolysis, its occurrence was due to the placement of prostheses and not a disease that induces bone resorption through its intrinsic physiopathology
Sedik et al. [67]	This study did not evaluate bone resorption
Xie et al. [68]	Although authors studied osteolysis, its occurrence was due to the placement of prostheses and not a disease that induces bone resorption through its intrinsic physiopathology
Yin et al. [69]	This study did not evaluate the use of extracellular vesicles
Zhang et al. [70]	The focus was not the control of bone resorption
Ren et al. [71]	This study did not evaluate the treatment of bone resorption
Ye et al. [72]	Study i*n vitro,* only
Gao et al. [73]	Although authors studied osteolysis, its occurrence was due to the placement of prostheses and not a disease that induces bone resorption through its intrinsic physiopathology
Li et al. [74]	Although authors studied osteolysis, its occurrence was due to the placement of prostheses and not a disease that induces bone resorption through its intrinsic physiopathology
Wang et al. [75]	Study i*n vitro,* only
Yang et al. [76]	The objective of this study was to compare the action of exosomes extracted from normal patients and patients with congenital pseudarthrosis of the tibia
Hayashi et al. [77]	The study used polyethyleneimine nanoparticles (PEI-NPs) and not extracellular vesicles (EVs)
Qayoom et al. [78]	The work did not focus on the study of EVs since no group was treated only with vesicles
Yun et al. [79]	The EVs were not extracted from cells, but from bovine colostrum
Cui et al. [80]	This study did not analyze the use of EVs in an animal model with bone resorption
Hu et al. [81]	The EVs were not extracted from cells, but from blood plasma
Xu et al. [55]	This study did not analyze the use of EVs in an animal model with bone resorption

As a result, studies were grouped by disease model. Table 2 described the osteoporosis and related conditions, Table 3 addressed periodontitis, and Table 4 included other diseases for which there were insufficient studies for grouping. Each disease type was analyzed separately, providing a comprehensive overview. Outcomes were similar across groups, and final results were based on all collected data. The same analytical method was used for molecular and structural endpoints. For further details, the respective disease tables can be consulted.

**Table 2:** Summary of characteristics and results from individual osteoporosis studies

Study	Animal specie and number	Disease associated with bone resorption	Extracellular vesicles					Groups	Analysis		Primary outcome	Secundary outcome
			size	extraction and characteriza-tion	origin-cell	dosage	administration		periods	types		
Kim et al. [20]	10-week-old female Sprague–Dawley rats (n = 48)	OVX-osteoporosis	< 100 nm (peak 77 nm)	Ctfr, Filtration, and TEM, NTA, proteome analysis, WB, ELISA	Human Amniotic Membrane Stem Cells	EVs (1 × 10*8, 3 × 10*8 or 1 × 10*9 particles/rat), ZA (100 ug/kg) or vehicle (PBS – 100 uL/rat)	Systemic injection (intravenously once a week for 8 weeks)	Sham, OVX, ZA, EVs (1 × 10*8), EVs (3 × 10*8) and EVs (1 × 10*9)	8 weeks	ELISA, biochemistruy analysis, dual energy X-ray, micro-CT, Biomechanical Measurement	EVs treatment (1 × 10^9) attenuated cancellous bone loss and restored bone mineral density and bone strength	Increase of 17β-estradiol, ALP, calcium, GFs, and osteogenesis after treatment (near-full recovery in the high-dose 1 × 10^9)
Chang et al. [25]	8-week-old female C57BL/6 mice (n = 40)	OVX-osteoporosis	150 nm	UCtfr, flow cytometry, WB, RT-PCR, and TEM	Mesenchymal stem cells	Antimouse RANKL antibody (5 mg/kg), EV (5 μg/μL) and EV@R (5 μg/μL). Sham and OVX injected with PBS	Systemic injection (intravenously injection through tail vein every 3 days for 8 weeks)	Sham, OVX, EV, EV@R and anti-RANKL	8 weeks	Micro-CT, EDTA, TRAP, IHC, IF, WB, RT-qPCR, flow cytometry	EV@R (EVs from MSCs overexpressing RANK) reversed osteoporosis conditions through inhibiting bone loss and promoting bone regeneration	Reduction of TRAP and RANKL, and increase of OCN, Osterix, Runx2, and COL-1 in EV@R group
Wang et al. [27]	3-month-old or 15-month-old male C57BL/6 mice (n = 30)	Senile osteoporosis	117.5 ± 32.5 nm (YO-EVs) and 107.5 ± 40.5 nm (SO-EVs)	UCtrf and TEM	young (YO) and senescent (SO) osteocytes	100 ug per ml of PBS	Systemic injection (in tail vein twice per week during 1 month)	Control, YO-EVs, and SO-EVs	4 weeks	HE, IHC, micro-CT, qRT-PCR, flow cytometry, proteomic analysis	Control of bone resorption and occurrence of bone regeneration more significant in group with YO-Evs therapy	Decrease of RANKL, TRAP and osteoclasts, and increase of ALP, OCN, and osteoblasts in YO-Evs therapy group
Xing et al. [28]	12-week-old male Sprague–Dawley rats (n = 24)	DO	30–130 nm	Ctfr and TEM, WB, NTA	Skeletal muscle tissues	0.8 mg/kg body weight	Systemic injection (in tail vein every other day for 8 times in total)	Sham, DN + PBS and DN + ES_-_Exo	8 weeks	HE, IHC, micro-CT, WB, qRT-PCR, ELISA	EVs promoted osteogenesis as well as suppress bone resorption	DN + ES-Exos enhanced OCN and Runx2, reduced positive staining of bone resorption marker CK
Huang et al. [30]	6-week-old male C57BL/6 J mice (n = 40)	DO	50–200 nm (114.3 nm ± 5.0 nm)	Ctrf and CSLM	Skeletal muscle tissue	200 ug of EVs in 200uL of saline	Systemic injection (intravenously injection twice per week for 3 weeks)	Sham, Control1, Control2, DS + EVs1, and DS + EVs2	4 weeks	Masson, IHC, IF, micro-CT, qRT-PCR, WB	Control of bone resorption and occurrence of bone regenerationmore significant in therapy with EV independently of DO etiology	Decrease of TRAP and osteoclastogenesis, and increase of Runx2, ALP, Osterix, COL-1 and osteoblasts when the EVs therapy was used
Yang et al. [35]	Female C57BL/6 mice (n = 24)	OVX-osteoporosis	30-150 nm	UCtrf and TEM	Macrophage (Mφ) cultived with or without SrBGNs	100 μg of EVs in 100 μl PBS	Local injection (intraperitoneally twice per week during 4 weeks)	Sham, PBS, Mφ-EVs e SrBGN + Mφ-Evs	4 weeks	HE, Masson, IHC, micro-CT, qRT-PCR, WB e ELISA	Control of bone resorption and occurrence of bone regeneration more significant in SrBGN + Mφ-Evs, group	Decrease of TRAP, RANKL, Th17, and osteoclasts, and increase of Treg, Runx2, OPN and osteoblasts
Yang et al. [36]	8-week-old female C57BL/6 mice (n = 30) and 12-week-old female Wistar rats (n = 12)	OVX-osteoporosis	100 nm	Ctrf and TEM	mc3t3-e1 cells	30 μg of EVs in mice, and 100 µg of EVs in rats	Local injection (in periosteal into the marrow cavity of the tibiae once a week during 8 weeks in mice; Injection in socket once a week during 8 weeks in rats)	MIce: Sham, DS + EVs, DS + EVs + EphA2, DS + EVs + METTL14, DS + EVs + EphA2 + METTL14 Rats: DS + ZOL, DS + EVs + METTL14, and DS + EVs + EphA2 + METTL14	8 weeks	HE, IHC, IF, micro-CT, WB, qRT-PCR	Bone regeneration, and absence of osteonecrosis in groups with EVs + METLL14	Inhibition of osteoclast differentiation via inhibition of NFATc1 in groups with EVs + METLL14
Wang et al. [40]	12-week-old female C57BL/6 mice (n = 40)	OVX-osteoporosis	40–100 nm	Ctrf and TEM	Mesenchymal Stem Cells (MSCs)	20 µL de MSC-Evs	Local injection (in femural periosteum twice at one week)	Sham, DS, DS + miR-27, DS + miR-27 mimic, DS + DMSO, DS + MSC-EVs, DS + MSC-Evs + miR-27 inibidor, DS + MSC-Evs + miR-27 inibidor + RNA-DKK2	1 week	HE, IHC, micro-CT, ELISA, WB, qRT-PCR	Control of bone resorption and occurrence of bone regeneration more significant in group with MSC-EVs therapy	Descrease of osteoclasts differentiation, and increase of ALP, OCN and Runx2 with MSC-EVs therapy miR-27a participation in DKK2 inibition
Yang et al. [41]	8-week-old female C57BL/6 mice (n = 40)	OVX-osteoporosis	40-130 nm	Ctrf and SEM (scanning electron microscope)	BMSCs modified or not with micro-nano bioactive glass (BGN)	100 µg de EVs in 100 µL of PBS	Local injection (intraperitoneally injected thrice a week for 4 weeks)	Sham, DS, DS + EVs e DS + BGN + EVs	4 weeks	HE, Masson, IHC, micro-CT, qRT-PCR, WB e ELISA	Control of bone resorption and occurrence of bone regeneration more significant in group with BGN + EVs therapy	Inhibition of osteoclasts, and normal indices of OPG in BGN + EVs group
Cui et al. [42]	12-week-old female C57BL/6 mice (n = 48)	OVX-osteoporosis	99.8 ± 34 nm and 118 + -42 nm	Ctrf and TEM	derivated of induzed pluripotent stem cells (iMSCs)	1.0 × 10^11^ particles in 100 μL PBS	Systemic injection (intravenously injected once a week for 6 weeks through the tail vein)	Sham, DS + PBS, DS + EVs (BT-Exo, Exo-siShn3, BT-Exo-siShn3, or BT-Exo-siCon)	2 months	HE, IF, RT-PCR, WB, ELISA	Control of bone resorption and occurrence of bone regeneration more significant in group with BT-Exo-siShn3 therapy	Increase of OPG, ALP, OCN and osteoblastic differentiation in BT-Exo-siShn3 group
Lee et al. [44]	8-week-old female mice (n = 24)	OVX-osteoporosis	88 nm	Filtration and TEM	Adipose tissue derived Stem Cells (ASCs)	ASC-EVs (1 × 10^8^ ou 5 × 10^8^ particles in 100 μl of PBS) or ASCs (5 × 10^5^ cells in 100 μl of PBS)	Systemic injection (intravenously injected; ASC-EVs, thrice a week for 2 weeks; ASCs once a week for 2 weeks)	Sham, DS + PBS, DS + ASCs, and DS + ASC-EVs	5 weeks	IHC, micro-CT, qRT-PCR, WB, ELISA,flow cytometry	Similar control of bone resorption in EVs and Cells groups	Decrease of RANKL, TRAP and osteoclast, and balance of OPG levels miR-21-5p participation in Acvr2a down-regulation
Xiao et al. [46]	6-month-old male C57BL/6 J mice (n = 30)	DOP	105 nm	Ctrf and TEM	BMSCs	5 mg/kg	Systemic injection (tail vein injections twice a week for 4 weeks of 0.1 mL per injection)	DS + PBS, DS + EVs, DS + CMS-EVs (cyclic mechanical stretch-exposed EVs)	4 weeks	HE, IHC, micro-CT	Control of bone resorption more significant in CMS-EVs group	Decrease of RANKL and actin of osteoclasts in CMS-EVs group
Zhang et al. [47]	8 and 10-week-old SD rat (n = 63)	Diabetic osteoporosis	40–100 nm	Filtration, Centrifugation and TEM	ASCs	1.6 mg/kg de EVs	Systemic injection (in tail vein every two days)	Sham, DS + PBS, DS + EVs	42 days	dual-energy X-ray, WB, qRT-PCR, ELISA	Control of bone resorption with EVs therapy	Decrease of pro-inflammatory cytokines and osteoclasts, and inhibition of inflammassome in EVs groups
Zhang et al. [48]	8 and 10-week-old SD rat (n = 75)	Diabetic osteoporosis	50-100 nm	UCtrf and TEM	Epididimal adipose tissue derived MSCs	1.6 mg/kg EVs (Exo, vector-Exo, or miR-146a-Exo)	Systemic injection (into the tail vein)	Sham, DS + PBS, DS + EVs (Exo, vector-Exo, or miR-146a-Exo)	12 semanas	dual-energy X-ray, WB, qRT-PCR, ELISA	Control of bone resorption with EVs therapy, especially in miR-146a-Exo group	Decrease of pro-inflammatory cytokines and osteoclasts, and inhibition of inflammassome in EVs groups miR-146a enhanced the inhibitory effect on bone resorption of osteoclasts
Hu et al. [49]	8-week-old female C57BL/6 mice (n = 30), 16-week-old male (n = 16) and 3-month-old mice (n = 30)	OVX-Osteoporosis, senile osteoporosis and DO	60-150 nm	Ctrf and TEM	Human umbilical cord MSCs	100 μg of EVs in 100 μl of PBS	Systemic injection (intravenously into the tail vein once a week during 2 or 3 months or OVX-and senile osteoporosis, respectively. For DO, the injection twice a week during 21 days)	Sham, DS + PBS, and DS + EVs	2 months (OVX) and 3 months (senile and DO)	HE, IHC, micro-CT, ELISA, qRT-PCR e WB	Control of bone resorption and occurrence of bone regeneration more significant in group with EVs therapy	Decrease of TRAP and osteoclasts, and increase of Runx2, COL-1 and osteoblasts
Chen et al. [52]	8 and 10 weeks-old female C57BL/6 mice (n = 30), 16-month-old mice (n = 16), 3-month-old mice (n = 31)	OVX-osteoporosis, senile osteoporosis, DO	50 nm	Ctrf and TEM	urine-derived stem cells (postmenopausal women)	100 µg de EVs	Systemic injection (into tail vein once a week during 2 and 3 months for OVX-and senile osteoporosis, respectively. For DO, the injection twice a week for 3 months)	Sham + PBS, DS + PBS, DS + EVs	2 months (OVX) and 3 months (senile and DO)	HE, IHC, micro-CT, WB, qRT-PCR, WB, ELISA	Control of bone resorption and occurrence of bone regeneration more significant in group with EVs therapy	Decrease of TRAP and osteoclasts, and Increase of OCN, ALP, Runx2, OPG and osteoblasts
Luo et al. [53]	3-month-old female C57BL/6 mice (n = 24) and 3-month-old male C57BL/6 mice (n = 12)	OVX-osteoporosis and bone fracture	35 a 105 nm	Ctrf and TEM	BMSCs	100µL of EVs	Systemic injection (into the tail vein once a week during 2 or 6 weeks for OVX or frature, respectively)	Sham + PBS, DS + PBS, DS + EVs, DS + EVs + Aptamer	2 months	IHC, micro-CT, qRT-PCR, flow cytometry	Bone regeneration more significant in group with EVs therapy; absence of influence in bone resorption	Increase of ALP, Runx2, and osteoblasts in EVs groups; levels of TRAP and osteoclasts did not modify with use of EVs
Song et al. [54]	8-week-old female C57BL/6 mice (n = 27)	OVX-osteoporosis	106–150 nm	UCtrf and TEM or NTA	Vascular Endothelial Cells	100 μg of EVs in 100 μL of PBS	Systemic injection (intravenous injection twice a week per 6 weeks)	Sham, Disease, and Disease + EVs	6 weeks	HE, IHC, micro-CT	Control of bone resorption and occurrence of bone regeneration more significat in EVs group	Decrease of pro-inflammatory cytocynes, TRAP, and osteoclasts in EVs group miR-155 participated reducing bone resorption

*NTA*: nanoparticle tracking analysis, Ctrf: centrifugation, *TEM*: Transmission electron microscopy, *LPS*: Lipopolysaccharides, *EVs*: Extracellular vesicles, *PBS*: Phosphate-Buffered Saline, *DS*: disease, *HE*: Hematoxylin and eosin, *IHC*: Immunohistochemistry, *IF*: Immunofluorescence, micro-CT: Micro computed tomography, *WB*: Western blot, *qRT-PCR/RT-PCR*: Real-time polymerase chain reaction, *RANKL*: Receptor activator of nuclear factor kappa-Β ligand, *TRAP*: Tartrate-resistant acid phosphatase, *UCtrf*: Ultracentrifugation, *ALP*: Alkaline phosphatase, *OCN*: Osteocalcin, *IL-10*: Interleukin-10, *Runx2*: Runt-related transcription factor 2, *OPN*: Osteopontin, *VEGF*: Vascular endothelial growth factor, *DO*: disuse osteoporosis, *CLSM*: confocal laser scanning microscope, *COL-1*: type 1 collagen, *OVX*: ovariectomy, SrBGNs: strontium doped bioactive glass nano-particles, *Sham*: un-manipulated animal, *Th17*: T helper 17 cell, *Treg*: regulatory T cell, *EphA2*: Ephrin type-A receptor 2—METTL14—methyltransferase 14, N6-adenosine-methyltransferase subunit, *ZOL*: Zoledronic acid, *TGF-beta 1*: transforming growth factor-beta 1, *DMSO*: dimethylsulfoxide, *TNF-α*: Tumor Necrosis Factor Alpha

**Table 3:** Summary of characteristics and results from individual periodontitis studies

Study	Animal specie and number	Disease associated with bone resorption	Extracellular vesicles					Groups	Analysis		Primary outcome	Secundary outcome
			size	extraction and characteriza-tion	origin-cell	dosage	administration		periods	types		
Cui et al.[17]	8-week-old male C57BL/6 J mice (n = 24)	Periodontitis	AE2Ns (157.9 nm), and Kae@AE2Ns (152.1 nm)	UCtfr, TEM, NTA	Macrophages M2 and macrophage-derived apoptotic vesicles	AE2Ns and Kae@AE2Ns (5 mg/kg)	Local injection (periodontal tissue every two days)	Control, ligature, AE2Ns and Kae@AE2Ns	10 days	Micro-CT, HE, Masson, IHC, IF, WB, qRT-PCR	The EVs were capable of mitigate periodontal inflammation, reduced the periodontal deterioration, and promote periodontal bone repair. The Kae@AE2Ns group exhibiting the most pronounced improvement	Increase of of ONC and COL1, and reducion of inflammatory cells
Fan et al.[18]	8-week-old male Sprague Dawley (n = 25)	Diabetic periodontitis	NS	UCtfr and TEM, SEM	Schwann cells	PGSP, SC Exo, and GSP@SC Exo (1 μg/μL, 100 μL) and Sham and control (equivalent PBS)	Local injection (periodontal pocket twice weekly for 2 and 4 weeks)	Sham, control, PGSP, SC Exo and GSP@SC-Exo	2 and 4 weeks	ELISA, qRT-PCR, micro-CT, HE, IHC	GSP@SC-Exo has effect on bone regeneration, promotes neovascularization. And immunoinflammatory modulation	Increase of OPN and OCN, M2 macrophage polarization, and alleviation of local inflammation and antioxidant and antibacterial capacity
Guo et al.[19]	4-week-old male Sprague Dawley rats (n = 26)	Diabetic periodontitis	Average size 120 nm	UCtfr and TEM	Tissue of antler blastema obtained from a healthy 2-year-old male sika deer	AnSC exos (50 μg/ml) and hBMSC-exos (50 μg/ml) and PBS (10uL)	Local injection (periodontal pocket on alternate days)	Normal, Ligation, Control, hBMSC-exo and AnSC-exo	3 weeks	Micro-CT, HE, IHC, IF, ARS, ALP, qRT-PCR, WB,	AnSC-Exo reduced effectively alveolar bone destruction and resorption under diabetes condition, and demonstrated significantly less inflammatory cells compared to other groups	Reduction of IL-1β, and inflammatory process in AnSC-exo group, as well as increase of IL-10
Lai et al.[21]	8-week-old male C57BL/6 mice (n = 24)	Periodontitis	50-150 nm	UCtfr and TEM, NTA, WB, RT-PCR	Macrophages DP7-C stimulated	10 µL of exosome suspension (1.2 µg/µL, containing 12 µg of exosomal protein) or 10 µL of PBS	Local injection (into the gingiva tissue at six distinct buccal and palatal sites every 3 days)	Control, Exo^Blank^, Exo^DP7−C^ and Exo^miR−21b^	14 days	Micro-CT, HE, IHC, dual-luciferase, qRT‒PCR, WB	Exo^miR−21b^ group demonstrated reduced alveolar bone loss, higher bone volume and bone mineral density, greater trabecular thickness and number, and New bone formation were detected at the alveolar crest area	Reduction of inflammatory infiltrates and osteoclast. Increase of Osterix in Exo^miR−21b^ group. Exosomal miR-21b targets SOCS1 to activate the JAK2/STAT3 signaling pathway
Li et al.[22]	7-weeks-old female C57BL/6 mice (n = 20)	Periodontitis	50-150 nm	Lyophilized-Evs, TEM, SEM, and DLS	Human Umbilical Cord Mesenchymal Stem Cells	50μL (hydrogel) and 50μL (exo@H = 1.2 × 1010 particles /mL)	Local injection (around mouse’s teeth twice a day during 8 days)	Sham, Model, Hydrogel and Exo@H	8 days	WB, HE, GO and KEGG, RT-qPCR, Luciferase Reporter Assay	Exo@H group revealed significant reduction in alveolar bone resorption and inflammatory response of periodontal tissues. There was no significant difference between model and hydrogel group	Decrease of TRAP and osteoclasts numbers, downregulation of miRNAs which modulates inflammatory responses pathways in periodontal tissue in Exo@H group
Liu et al.[23]	6-week-old male C57BL/6 mice (n = 20)	Periodontitis	NC-EVs (130 ± 50.0 nm) and EPO-EVs (131 ± 50.9 nm)	Ctfr UCtfr, and TEM, NTA	Erythropoietin stimulated macrophage	100uL hydrogel, NC-EVs/hydrogel, EPO-EVs/hydrogel (100uL) or PBS (100uL)	Local injection (once a week for 3 weeks)	Control, Hydrogel, NC-EVs/hydrogel and EPO-EVs/hydrogel	3 weeks	qPCR, WB, micro-CT, IF, HE, dual-luciferase assay	EPO-EVs/hydrogel effectively alleviated the destruction of alveolar bone in the experimental periodontitis mouse model	Increase of OCN in EPO-EVs/hydrogel treatment. miR-5107-5p promotes alveolar bone regeneration via the EGFR/RhoA axis in EPO-EVs group
Rojas et al.[24]	C57BL/6 wild-type mice (N = NS)	Periodontitis	100–200 nm (mean size 141 nm ± 71 nm). Other three small fractions were observed, ranging from around 288–506 nm	UCtfr and TEM or NTA	Regulatory T cell (Treg)	1 × 10^8^ or 2.5 × 10^8^ RATEVs in 5µL of PBS. Sham injections with PBS as RATEVs-free control were used	Local injection (palatal periodontal mucosa to the second molars at day 3 and 6 after ligature)	Healthy, Perio, Perio + RATEVs-lo and Perio + RATEVs-hi	10 days	WB, flow cytometry, TRAP, EDTA	RATEVs inhibit osteoclastogenesis and ameliorate alveolar bone loss during experimental periodontitis	Reduced TRAP, RANKL, CD4 + Foxp3 + Tregs, IL-17A, and osteoclast number in the groups RATEVs. Increase of CD73 expression in RATEVs group
Huang et al. [26]	8-week-old male C57BL/6 mice (n = 18)	Periodontitis	130 nm (NTA) or 30–200 nm (TEM)	Ctrf and TEM	LPS-dental follicle cells	100 ug of Evs per ml of PBS	Local injection (in periodontal tissue on 3 times at 2-day intervals over one week)	Control, DS, and DS-EVs	2 weeks	HE, IHC, IF micro-CT, WB, qRT-PCR, flow cytometry	Control of bone resorption more significant in group with EVs therapy	Decrease of RANKL, TRAP and osteoclasts, and increase of M2-type macrophage in EVs group
Lu et al. [31]	6 and 8-week-old male mice (n = 24)	Periodontitis	50–150 nm	Ctrf and TEM	Periodontal ligament stem cells	300 ug of Evs	Local injection (in periodontal tissue once a week during 4 weeks)	Control, DS, DS-EVs1 (normal glucose), and DS-EVs2 (high glucose)	4 weeks	HE, micro-CT, RT-PCR, WB	Control of bone resorption and occurrence of bone regeneration more significant in therapy with EV cultured in normal glucose conditions	Downregulation of genes involved in osteoclastogenesis was more significant when EVs were extracted from stem cells and cultured in normal glucose conditions MiR-31-5p participation on eNOS inhibition
Luo et al. [32]	8-week-old male SD rats	Periodontitis	40–120 nm	UCtrf and TEM	CXCR4-overexpressing 293 T/17 cells	300 ug	Local injection (into the periodontium of the second maxillary molar)	Control, DS-EVs, DS-CXCR4-miR126-EVs, miR126-EVs	2 weeks	HE, IHC, micro-CT, WB, qRT-PCR, ELISA	Control of bone resorpotion more significant in CXCR4-miR126-Evs group	Descrease of pro-inflammatory cytocines, TRAP, M1-type macrophage and osteoclasts, and increase of IL-10, and M2 type macrophage in CXCR4-miR126-Evs group miR-126 participation in p48 MAPK signaling pathway
Pan et al. [33]	6 and 8-week-old male C57BL/6 mice (n = 24)	Periodontitis	30–200 nm	UCtrf and TEM	Osteoclasts	4ug of Evs	Local injection (10 μL of PBS or OC-EVs in second molar at 0, 4, 8, and 12 days)	Control, DS-PBS, and DS + EVs	14 days	HE, IHC, micro-CT, qRT-PCR, WB	Absence control of bone resorption and bone regeneration when EVs from osteoclasts were used	Decrease of Runx2, ALP and osteoblasts, and increase of pro-inflammatory cytokines, TRAP and osteoclasts miR-5134-5p participation in JAK2/STAT3 axis
Qiao et al. [34]	8-week-old male SD rats (n = 24)	Periodontitis	30–150 nm (average size de 84.06 nm)	UCtrf and TEM	Dental Pulp Stem Cells	50 ug of EVs per mL of PBS	Local injection (10µL in molars daily per 30 days)	Control, DS, DS + PBS, and DS + EVs	30 days	HE, IHC, micro-CT, qRT-PCR, WB, flow cytometry	Control of bone resorption and occurrence of bone regeneration more significant in EVs group	Decrease of pro-inflammatory cytokines, M1 types macrophages, and osteclasts, and increase of OCN, OPN, COL-1, and M2 type macrophage
Chen et al. [37]	6 and 8-week-old C57BL/6 mice	Periodontitis	30–150 nm	UCtrf and TEM	M2 type macrophage	50 or 100 µg of EVs in mL of PBS	Local injection (Periodontal)	DS + PBS, DS + EVs1, DS + EVs2	2 weeks	HE, IHC, micro-CT, ELISA, flow cytometry	Control of bone resorption and occurrence of bone regeneration more significant in EVs groups, especily in high concentration	Descrease of TRAP and osteoclasts, and increase of Runx2, ALP, OCN, and IL-10 in EVs groups
Shimizu et al. [38]	9 and 10-week-old male C57BL/6 J rats (n = 30)	Periodontitis	66–110 nm	UCtrf and NTA	human leukocyte antigen (HLA) haplotype homo dental pulp cells (HHH-DPCs)	5µL of EVs (7.5 × 10^8^ particles)	Local injection (at 0, 2 and 4 days after periodontal ligature)	DS, DS + saline, and DS + EVs	7 days (3 days after the end of applications)	HE, IHC, WB, qRT-PCR, micro-CT	Control of bone resorpotion more significant in EVs group	Decrease of RANKL, and osteoclasts in EVs group
Nakao et al. [45]	8-week-old female C57BL/6NCrSLc mice (n = 20)	Periodontitis	109 ± 3.1 nm and 104 ± 1.8 nm	Ctrf and TEM	Gingival tissue derived MSCs	20 μg of EVs in 20 μL de PBS	Local injection (into the palatal gingiva)	Control-PBS, DS + PBS, DS + EVs, DS + EVs-TNF-α	7 days	IHC, RT-PCR, WB	Control of bone resorption more significant in group with EVs therapy, especially in EVs-TNF-α group	Decrease of M1-type macrophages, TRAP, RANKL, and osteoclasts, and increase of OPG and M2-type macrophage in EVs groups, especially in group with TNF-α miR-1260b targeting Wnt5a expression and JNK signaling pathway
Wei et al. [50]	9 and 10-month-old C male D-1 mice	Periodontitis	100 nm	UCtrf and TEM	Stem cells from human exfoliated deciduous teeth	20 μg of EVs	Local injection (periodontal injection once a week during 2 weeks)	Control, DS, DS + PBS, DS + Cell, and DS + EVs	4 weeks	HE, Masson, IHC, qRT-PCR, WB and flow cytometry	Control of bone resorption and occurrence of bone regeneration more significant in group with EVs therapy	Decrease of pro-inflammatory cytokines, and increase of ALP, Runx2, and osteogenic differenciation

*NTA*: nanoparticle tracking analysis, *Ctrf*: centrifugation, *TEM*: transmission electron microscopy, *LPS*: lipopolysaccharides, *EVs*: extracellular vesicles, *PBS*: phosphate-buffered saline, *DS*: disease, *HE*: hematoxylin and eosin, *IHC*: immunohistochemistry, *IF*: immunofluorescence, *micro-CT*: micro computed tomography, *WB*: western blot, *qRT-PCR/RT-PCR*: real-time polymerase chain reaction, *RANKL*: receptor activator of nuclear factor kappa-Β ligand, *TRAP*: tartrate-resistant acid phosphatase, *UCtrf*: ultracentrifugation, *ALP*: alkaline phosphatase, *OCN*: osteocalcin, *IL-10*: interleukin-10, *Runx2*: runt-related transcription factor 2, *OPN*: osteopontin, *VEGF*: vascular endothelial growth factor, *DO*: disuse osteoporosis, *CLSM*: confocal laser scanning microscope, *COL-1*: type 1 collagen, *OVX*: ovariectomy, *SrBGNs*: strontium doped bioactive glass nano-particles, *Sham*: un-manipulated animal, *Th17*: T helper 17 cell, *Treg*: regulatory T cell, *EphA2*: ephrin type-A receptor 2—METTL14—methyltransferase 14, N6-adenosine-methyltransferase subunit, *ZOL*: Zoledronic acid, *TGF-beta 1*: transforming growth factor-beta 1, *DMSO*: dimethylsulfoxide, *TNF-α*: tumor necrosis factor alpha, *NS*: not specified, *DLS*: dynamic light scattering, *KEGG*: kyoto encyclopedia of genes and genomes, *GO*: gene ontology

**Table 4:** Summary of characteristics and results from individual studies of other diseases

Study	Animal specie and number	Disease associated with bone resorption	Extracellular vesicles					Groups	Analysis		Primary outcome	Secundary outcome
			size	extraction and characteriza-tion	origin-cell	dosage	administration		periods	types		
Yuan et al. [29]	8 and 10-week-old male SD rats (n = 60)	Steroid-induced osteonecrosis	40–150 nm	UCtrf and TEM	M2-type macrophage	100 ug per ml of PBS	Systemic injection (intravenously once a week during 4 weeks)	control, PBS, and EVs therapy	4 weeks	HE, IHC, micro-CT, WB, qRT-PCR	Control of bone resorption and occurrence of bone regeneration more significant in group with EVs therapy	Descrease of pro-inflammatory cytokines, TRAP and M1-type macrophage, and increase of M2-type macrophage, IL-10, ALP, Runx2, OPN, OCN, VEGF
Wang et al. [39]	8-week-old male C57BL/6 mice (n = 48)	Osteoartritis	30–150 nm	Exossome extraction kit and NTA	Bone Marrow Stem Cells (BMSCs)	10 µL of Evs per mL of saline	Local injection (in joint cavity everyday per 8 weeks)	Sham, Saline, EVs, and EVs + TGF-beta1	4 and 8 weeks	HE, IHC, IF, RT-PCR, WB, micro-CT	Control of bone resorption and occurrence of bone regeneration more significant in group with EVs-TGFbeta therapy	Descrease of TRAP, RANKL and osteoclastogenesis when the EVs-TGFbeta therapy was used miR-135b participation in MAPK6 pathway
Guo et al. [43]	Male rats (n = 20)	Mandibular hypoplasia (GATA-4 knockout)	100 nm	Ctrf and TEM	Orofacial stem cells	2 mg of EVs per ml of PBS	Local injection (two injection in periosteum of mandibular molar region every 3 days until 21 days)	control (PBS), and EVS	21 days	HE,micro-CT, qRT-PCR e WB	Control of bone resorption and occurrence of bone regeneration more significant in group with EVs therapy	Increase of OPG, OCN, OPN, Runx2 and decrease of RANKL and activation of osteoclast when EVs were used miR-206-3p participation on NFATc1 regulation
Zhang et al. [51]	Female SD rats with 200-300 g (n = 60)	Glucocorticoid-induced osteonecrosis	20-100 nm	UCtrf and TEM	Human MSCs	100 uL of EVs in 200 uL of PBS	Systemic injection (in tail vein daily for three weeks)	Sham, Disease, Disease + EVs, and Disease + EVs + miR-135b	3 semanas	IHC, micro-CT, WB, qRT-PCR, flow cytometry	Control of bone resorption and occurrence of bone regeneration more significant in group with EVs therapy, especially in EVs + miR-135b group	Decrease of apoptosis to osteocytes, and increase of OCN and osteoblasts in EVs therapy groups miR-135b regulated PDCD4, caspase-3 and OCN

*NTA*: nanoparticle tracking analysis, *Ctrf*: centrifugation, *TEM*: transmission electron microscopy, *LPS*: lipopolysaccharides, *EVs*: extracellular vesicles, *PBS*: phosphate-buffered saline, *DS*: disease, *HE*: hematoxylin and eosin, *IHC*: immunohistochemistry, *IF*: immunofluorescence, *micro-CT*: micro computed tomography, *WB*: western blot, *qRT-PCR/RT-PCR*: real-time polymerase chain reaction, *RANKL*: receptor activator of nuclear factor kappa-Β ligand, *TRAP*: tartrate-resistant acid phosphatase, *UCtrf*: ultracentrifugation, *ALP*: alkaline phosphatase, *OCN*: osteocalcin, *IL-10*: interleukin-10, *Runx2*: runt-related transcription factor 2, *OPN*: osteopontin, *VEGF*: vascular endothelial growth factor, *DO*: disuse osteoporosis, *CLSM*: confocal laser scanning microscope, *COL-1*: type 1 collagen, *OVX*: ovariectomy, *SrBGNs*: strontium doped bioactive glass nano-particles, *Sham*: un-manipulated animal, *Th17*: T helper 17 cell, *Treg*: regulatory T cell, *EphA2*: ephrin type-A receptor 2—METTL14—methyltransferase 14, N6-adenosine-methyltransferase subunit, *ZOL*: zoledronic acid, *TGF-beta 1*: transforming growth factor-beta 1, *DMSO*: dimethylsulfoxide, *TNF-α*: tumor necrosis factor alpha

### Study Risk of Bias Assessment and Reporting Bias Assessment

The risk of bias and the quality of scientific evidence were assessed using SYRCLE's RoB tool, specifically designed for animal studies. This tool consists of 10 items, subdivided into six categories: selection bias, performance bias, detection bias, attrition bias, reporting bias, and other sources of bias. For each of the ten items in this tool, two independent researchers (FMM and VAFM) read the full text article and assigned one of three concepts: affirmative (yes), negative (no), or uncertain (?). These concepts designate low risk of bias, high risk of bias, and unclear data, respectively. Any disagreements were resolved through discussion and agreement with another researcher (GGM), who reread the article and searched for the inconclusive item. The risk of bias for each study was qualitatively assessed by individually observing each item, without applying different weights to them.

## Results

### Study Selection

**Fig. 1:**
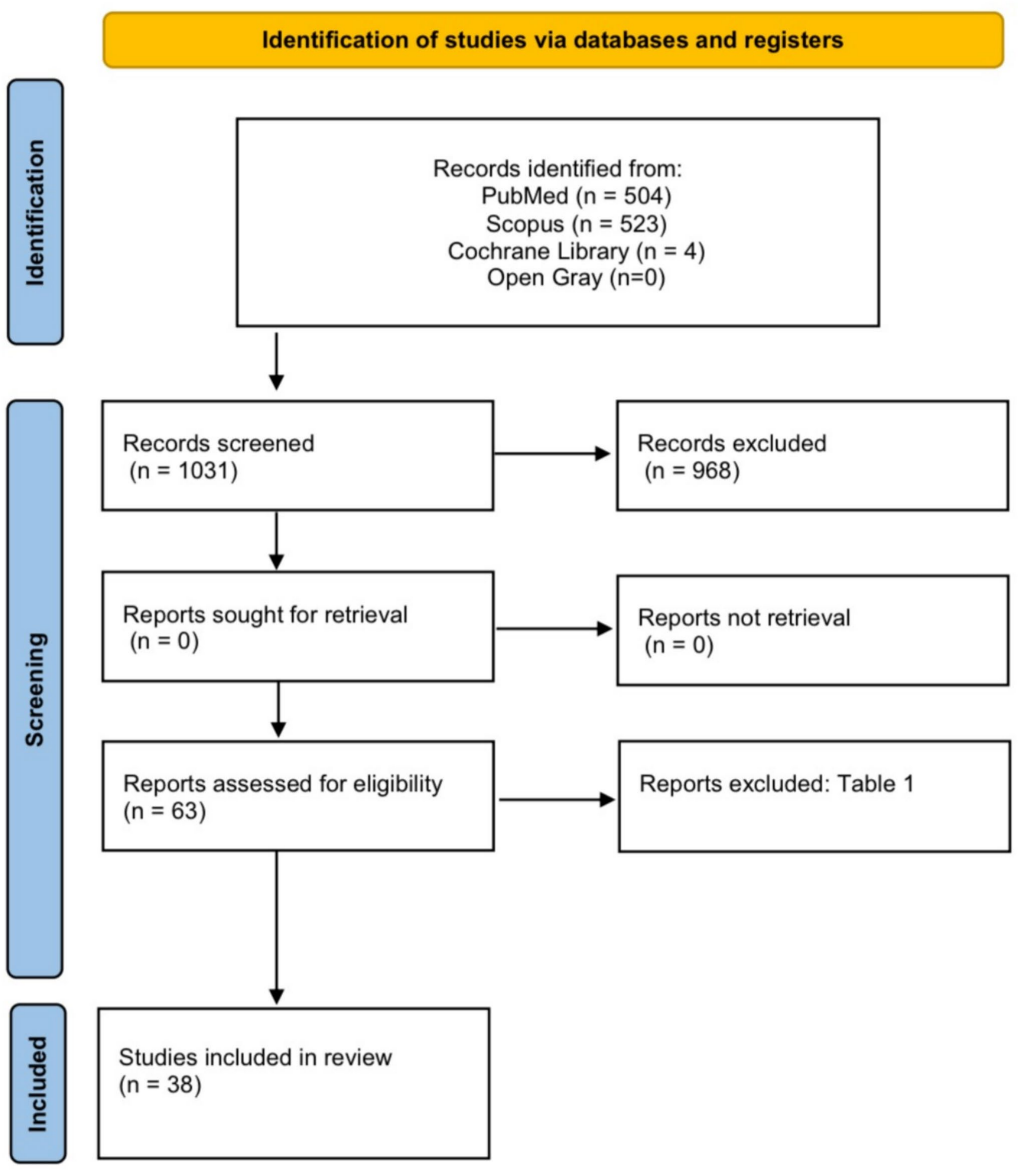
PRISMA 2020 flow diagram for new systematic reviews which included searches of databases and registers only. From: Page MJ, Mackenzie JE, Bossuyt PM, et al. The PRISMA 2020 statement: an update guideline for resporting systematic reviews. BMJ 2021;372: n. 71. https://doi.org/10.1136/bmj.n71

A total of 1031 titles and abstracts of articles were analyzed in cited databases, and after applying the eligibility criteria and excluding articles in duplicate, 63 studies were selected (Fig. 1). The Kappa test conducted between the two researchers in the systematic search for databases showed almost perfect since the agreement level was 0.866. After completing the reading and analysis of selected studies, 25 articles were excluded, totaling 38 studies included in our systematic review. Explanations for such exclusions can be seen in Table 1.

### Characterization of Studies

All included studies analyzed the control of bone resorption in animal models [17–54]. Bone resorption was induced by different diseases, including osteoporosis [20, 25, 27, 28, 30, 35, 36, 40–42, 44, 46–49, 52–54], periodontitis [17–19, 21–24, 26, 31–34, 37, 38, 45, 50], steroid-induced osteonecrosis [29, 51] osteoarthritis [39], and mandibular hypoplasia [43].

A total of 736 mice and 415 rats were used to evaluate the inhibition of the resorptive process in osteoporosis [20, 25, 27, 28, 30, 35, 36, 40–42, 44, 46–49, 52–54], being 397 mice [25, 35, 36, 40–42, 44, 49, 52–54] and 60 rats [20, 36] with ovariectomy/postmenopausal osteoporosis, 62 mice with senile osteoporosis [27, 29, 49, 52], 155 mice with osteoporosis by disuse [28, 30, 46, 49, 52] and 138 rats with diabetic osteoporosis [47, 48]. Regarding the evaluation of periodontitis, 204 mice [17, 21–23, 26, 31, 33, 38, 45] and 75 [18, 19, 34] rats were used [20, 22, 23, 27, 34, 55]; Luo et al., 2023 [32] did not mention the exact number of rats used, while Rojas et al. [24] and Chen et al. [37] did not accurately determine the number of mice. From another perspective, Wei et al. [50], 2020 cited only the range of animals used in their study, with the sample size being between 4 and 6 rats per group, without precision in the values. For other defects where EVs where tested, 12 mice were used in the bone fracture model associated with osteoporosis [53], 120 rats for defects arising from steroid-induced osteonecrosis [29, 51], 48 mice to evaluate osteoarthritis [39], and 20 rats for investigation of mandibular hypoplasia [43].

In general, EVs were extracted from origin cells using centrifugation [23, 26, 28, 30, 31, 36, 40–43, 45–47, 49, 52, 53], ultracentrifugation [17–19, 21, 23–25, 27, 29, 32–35, 37, 38, 48, 50, 51, 54] or filtration [20, 44, 47]. However, exceptions can be observed in the studies by Li et al. [22] which used the lyophilized EVs; Wang et al. [39], which used an exosome extraction kit, and Lee et al. [44], 2021, which used a multi-filtration system based on tangential flow filtration for the extraction of EVs. Most studies used transmission electron microscopy (TEM) to characterize extracellular vesicles [17–29, 33–37, 40, 42–54]. In addition, some studies used Nanoparticle tracking analysis (NTA) [17, 20, 21, 23, 24, 38, 39, 54], Scanning Electron Microscopy (SEM) [18, 22, 41], Confocal Laser Scanning Microscope (CLSM) [30], and Dynamic Light Scattering (DLS) [22]. All studies used the small extracellular vesicles [17–54].

Principally, the EVs were extracted from mesenchymal stem cells (MSCs) [19, 20, 22, 25, 34, 39–49, 51], especially those derived from bone marrow [19, 39, 41, 46, 53]. Mesenchymal cells from human amniotic membrane [20], gingival tissue [45], orofacial tissue [43], adipose tissue (ASCs) [44, 47], dental pulp [34], epididymis [48], and umbilical cord tissue (UC-MSCs) [22, 49] were also used for extraction. Chang et al. [25], Wang et al. [40], and Zhang et al. [51] removed the EVs from mesenchymal cells but did not specify the type, and Cui et al. [42] obtained the extracellular vesicle from induced mesenchymal stem cells. Other origin cells were used, such as macrophages [17, 21, 23, 29, 35, 37], regulatory T cells [24], dental pulp [38], exfoliated primary teeth [50], skeletal muscle [28, 30], Schuwann cells [18], and multipotent progenitor cells [19]. Furthermore, several other cell types were included as origin cells, and specific information about each can be seen in Tables 2, 3 and 4.

Regarding the posology of EVs in the intervention group, there was no constant value, with a wide variation in both dose and route of administration. Furthermore, EVs were injected locally [35, 36, 40, 41] or systemically [20, 25, 27, 28, 30, 42, 44, 46–49, 52–54], associated with vehicles (PBS or saline) for osteoporosis therapy. For periodontitis, the EVs were administrated via local injection into periodontal tissue in all studies [17–19, 21–24, 26, 31–34, 37, 38, 45, 50]. Systemic administration of EVs was performed for steroid-induced osteonecrosis [29, 51], and local administration was made for osteoarthritis [39], and mandibular hypoplasia [43]. Specific information concerning the posology for each study included in this review can also be seen in Tables 2, 3 and 4.

To assess bone resorption control, regeneration, and the involved molecular mechanisms, the studies utilized histological, image, and molecular analyses. In histological analysis, the tissue was stained or marked with hematoxyline-eosine [17–19, 21–23, 26–29, 31–43, 46, 49, 50, 52, 54], Masson’s trichrome [17, 30, 35, 41, 50], immunohistochemistry [17–19, 21, 25–30, 32–41, 44–46, 49–54] and immunofluorescence [17, 19, 23, 25, 26, 30, 36, 39, 42]. The studies evaluated the quality and quantity of bone with the aid of micro-CT [17–23, 25–41, 43, 48, 49, 51–54] or dual energy X-ray [20, 47, 48] for image analysis. Considering the molecular analysis, the studies used real-time polymerase chain reaction (qRT-PCR/RT-PCR) [17, 19, 21–23, 25–36, 38–44, 46–53], Western-Blot [17, 19–27, 29–52], flow cytometry [18, 20, 24–27, 34, 37, 44, 50, 51, 53], enzyme-linked immunosorbent assay (ELISA) [18, 20, 28, 32, 35, 37, 40–42, 44, 47–49, 52], proteomic analysis [20, 27], and luciferase assay [21–23].

In Tables 2, 3, and 4, findings can be observed about the studied diseases, EVs (type, origin cell, extraction and characterization), posology, experimental groups, periods and analysis, as well as data about the primary and secondary outcomes.

### Risk of Bias

The risk of bias was assessed using SYRCLE's RoB tool, considering six categories: selection bias, performance bias, detection bias, attrition bias, reporting bias, and other sources of bias. Selection bias included domains 1 to 3, which encompassed sequence generation, baseline characteristics, and allocation concealment, respectively. Thus, high scientific quality was observed for domains 1 and 2 in 29 [17–19, 21–24, 26–30, 32, 34, 36, 39, 40, 42, 44–54] and 31 [17, 19–29, 31–36, 39–42, 44–47, 49, 51–54] studies; however, for domain 3, all studies showed an unclear risk of bias because it was not possible to determine how, or whether, allocation concealment was performed [17–54].

Regarding performance bias, domain 4, which refers to random housing, showed low and unclear risk of bias in 23 [17–22, 24–29, 31, 34, 39, 40, 42, 46–48, 51, 54] and 15 [23, 30, 32, 35–38, 41, 43–45, 49, 50, 52, 53] studies, respectively. For blinding in domain 5, it was not possible to determine whether blinding occurred during the investigation, as no study clearly reported this information [17–54].

Detection bias, involving randomization and blinding in outcome assessment, demonstrated unclear risk of bias for most studies, as only 6 [29, 31, 34, 50, 52, 53] and 3 [29, 30, 52] studies provided information on these aspects in domains 6 and 7, respectively. Domain 8, referring to attrition bias, showed a low risk of bias for nearly all studies [17–37, 39–51, 53, 54], with the exception of two studies that presented a high risk of bias [38, 52]. Domain 9, addressing reporting bias, showed a low risk of bias for all studies [17–54]. In domain 10, other sources of bias, only three studies showed a high risk of bias [37, 38, 52], while the majority presented a low risk [17–36, 39–51, 53, 54].

**Fig. 2:**
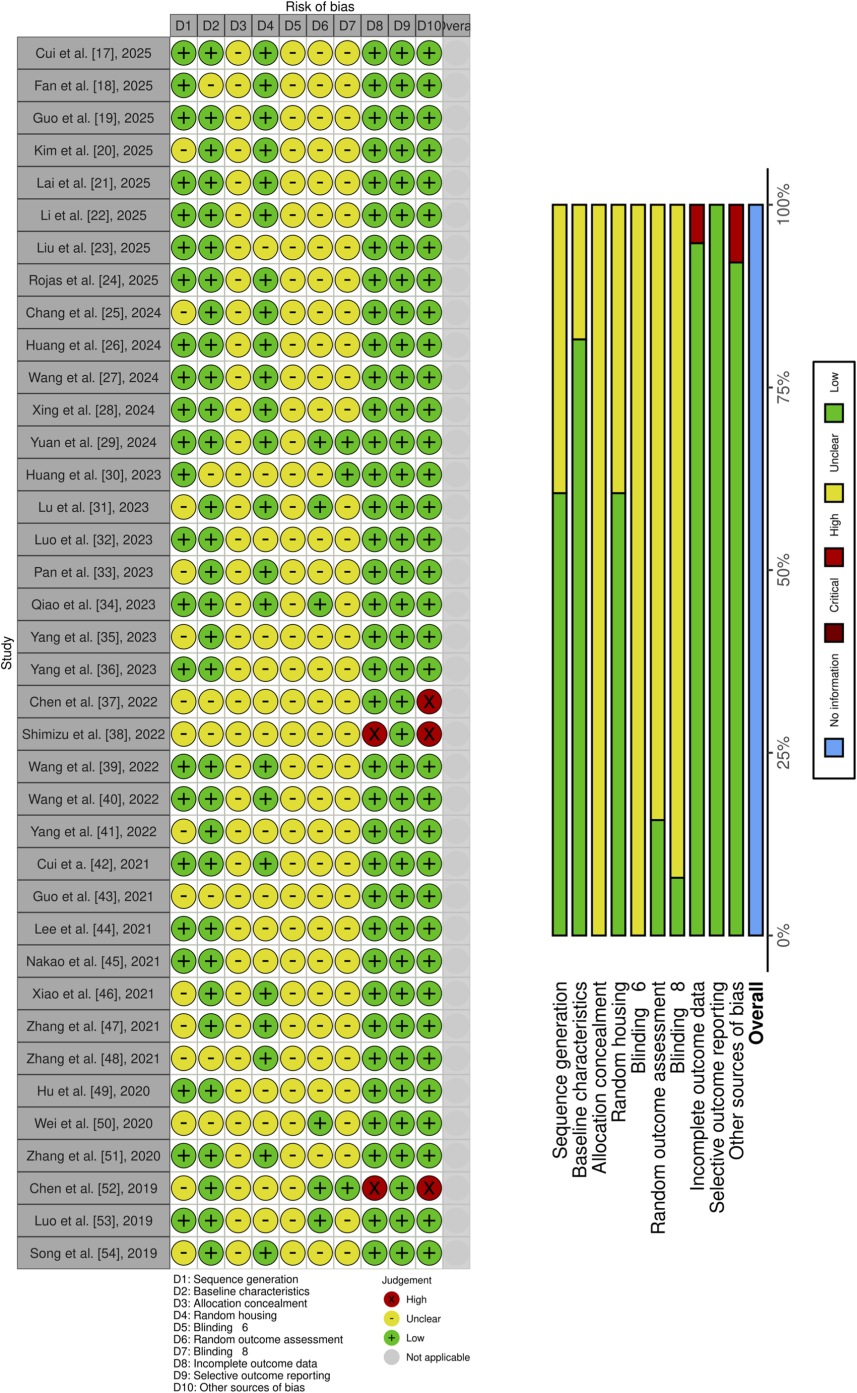
Risk of bias based on SYRCLE´s ROB tool represented by Risk-of-bias VISualization (Robvis)

The risk of bias for each study was qualitatively assessed by individually observing each item, without applying different weights to them, and the data were entered into a pre-formulated table that summarized the results and generated Fig. 2.

### Results of Individual Studies

The majority of studies demonstrated that EVs are promising in controlling bone resorption-related diseases in vivo [17–32, 34–52, 54]. Also, some of these studies evaluated the influence of EVs in promoting bone regeneration [18–25, 27–31, 34–37, 39–41, 43, 49–54], collaborating with the resolution of bone loss. Some studies have shown that functionalized or modified EVs are more effective for both actions compared to un-modified EVs [17, 18, 21, 23, 25, 32, 35, 36, 39, 41, 42, 45, 46, 48, 51].

Lee et al. [44], in 2021, verified similar effects to control of bone resorption when adipose tissue-derived stem cells (ASCs) or ASCs-EVs were used for therapy to osteoporosis. Luo et al. [53], in 2019, demonstrated EVs extracted from bone marrow stem cells promoted bone regeneration, but they did not influence bone resorption. Pan et al. [33], in 2023, using EVs extracted from osteoclasts, verified the absence of control of bone resorption and regeneration. And, Wang et al. [27], in 2024, verified senescent osteocytes were not able to control bone resorption in senile osteoporosis, and Lu et al. [31], in 2023, demonstrated that EVs extracted in high glucose conditions were not efficient to control bone resorption and promote bone regeneration.

Considering the secondary outcome of studies demonstrated the efficacy of EVs to control bone resorption, it was observed the significant reduction of TRAP [22, 24–30, 35, 37, 39, 44, 45, 49, 52, 54], RANKL [25–27, 35, 38, 39, 43–46], pro-inflammatory cytokines [19, 22, 24, 29, 32, 34, 47, 48, 50, 54], as well as inhibition or controlling on osteoclasts [19, 21, 26, 27, 30–32, 34–41, 43, 44, 46–49, 52, 54], M1-type macrophages [29, 32, 34, 45], and Th17 [35].

The molecular mechanisms involved in bone regeneration that collaborate with the boost of bone mass and control of bone resorption highlight the increase of molecules and growth factors essential to osteoblast differentiation. Thus, the presence of factor of runt-related transcription 2 (Runx2) [25, 28–30, 35, 42, 49–51, 53], alkaline phosphatase (ALP) [25, 27, 29, 30, 37, 40, 42, 50, 52, 53], osteocalcin (OCN) [17, 18, 23, 25, 27–29, 34, 37, 40, 42, 43, 51, 52], osteopontin (OPN) [18, 29, 34, 35, 52], type 1 collagen (COL-1) [17, 25, 30, 34, 49], Osterix [21, 25, 30]. Besides these aspects, osteoblast differentiation also influenced positively bone health in some studies [20, 27, 30, 35, 42, 49–51, 53]. An increase of OPG [41–44], regulatory T cell [35], Interleukin-10 (IL-10) [19, 29, 32, 47], and M2-type macrophage [18, 26, 32, 34, 45] is important to the regulation of bone resorption. It is also important to highlight the presence of vascular endothelial growth factor (VEGF), essential for angiogenic differentiation and consequent bone regeneration was noted by Kim et al. [20], and Wang et al. [27].

It was possible to highlight that the BMSCs-EVs were able to reduce RANKL [39, 46], TRAP [39], and pro-inflammatory cytokine (IL-1) [19], thereby controlling bone resorption [19, 39, 41, 46]. These studies also demonstrated bone regeneration [39, 41, 53], restored the normality of OPG levels [41], and showed increased level of IL-10, an immunosuppressive cytokine [19]. The UC-MSCs-EVs and ASCs decreased the number of osteoclasts [22, 44, 47] and levels of TRAP [22, 44, 49], inflammatory cytokines [47], and RANKL [44], as well as increased the number of osteoblasts [49], and level of COL1 [49], Runx2 [49], and OPG [44].

Also, the EVs obtained from macrophages controlled the bone resorption and increased bone regeneration [21, 23, 29], due decrease in osteoclast activity [21, 29, 35, 37] and improvement of Runx2 [29, 35, 37], OCN [17, 23, 29, 35], OPN [29, 37], Osterix [21], COL-1 [17] e IL-10 [29, 37] levels. The EVs isolated from skeletal muscle enhanced OCN [28], Runx2 [28, 30], Osterix [30], COL-1 [30], and osteoblasts [30], and controlled the bone resorption [28, 30].

For the other origin cells used for extraction and included in this review [18, 20, 24–27, 31–34, 36, 38, 40, 42, 43, 45, 48, 50–52, 54], despite the beneficial effects obtained in most studies, the absence of multiple studies for specific type of cells using the same EV origin prevented a comparative analysis of findings. Details about the primary and secondary outcomes were summarized in Tables 2, 3 and 4.

Besides that, the studies demonstrated that vesicles extracted from identical origin cells, even if utilized in different dosages, were efficient and promoted similar effects and outcomes, suggesting the potential to use lower doses of EVs for the treatment of diseases related to bone resorption.

Likewise, some authors have also evaluated the biodistribution of vesicles in different organs [23–25, 27, 35, 41, 42, 44, 52–54]. The main organs evaluated were bones [24, 25, 27, 35, 41, 42, 44, 52–54], more specifically the femur [35, 41, 42, 52, 54] and the tibia [42, 52], as well as liver [23–25, 35, 41, 42, 44, 52, 54], kidneys [23–25, 35, 41, 42, 44, 52, 54], spleen [23, 25, 35, 41, 42, 44, 52, 54], lungs [23, 25, 35, 41, 42, 44, 52, 54], heart [23, 25, 35, 42, 44, 52, 54], and lynphonods [24]; Wang et al. [27], Lee et al. [44], and Luo et al. [53], 2019 did not specify the bone evaluated; Cui et al. [42], and Chen et al. [52] even evaluated the brain. Overall, the liver showed the highest concentration of vesicles [35, 41, 42, 44, 52, 54], but some studies also reported significant vesicle signals originating from the bone [27, 35, 44, 52, 53], particularly from the trabecular surface [52]. Cytotoxicity was also assessed, revealing the absence of toxicity when using EVs [29, 30, 34, 43, 45–47].

## Discussion

The PICO question of the present systematic review "Would extracellular vesicle therapy be efficient for treating bone resorption?" was interpreted as having an affirmative answer, since the majority of studies have confirmed successful control bone resorption after using EVs, regardless of the disease responsible for this problem [17–32, 34–52, 54].

Despite five articles showing data indicating the absence of superiority of EVs therapy, the affirmative above remains possible. One study demonstrated similar and promising results when EVs or stem cells were used for treating bone resorption [44]. Due to difficulties in accessing stem cells and their possible immunological limitations, such as immune rejection in genetically different individuals [56], and the availability and low risk of immune rejection when using EVs [26–32, 34–43, 45–52, 54], making EV therapy a preferred choice.

EVs removed from senescent osteocytes [27] or extracted in high glucose conditions [31] were inefficient in controlling bone resorption. In contrast, EVs from young osteocytes or extracted in normal glucose conditions demonstrated effectiveness in this regard. Therefore, the condition of the EVs requires to be carefully assessed for clinical applications [27, 31]. Although Luo et al. [53] demonstrated EVs did not interfere in bone resorption, these vesicles stimulate bone regeneration, which benefits the imbalance of bone remodeling.

The unique study reporting an unsatisfactory outcome for controlling bone resorption was that of Pan et al. [33], in which EVs were derived from osteoclasts. Since EVs have the similar biological functions of their origin cells [9–11, 13], the ineffectiveness may be justified considering the intrinsic bone-resorptive function of osteoclasts [1–6]. Therefore, the appropriate choice of the cell of origin for EV extraction is essential for future clinical approaches.

The satisfactory effect of EVs can be explained when the molecular mechanisms are analyzed carefully. In the present review, it was observed EVs were able to decrease levels of TRAP [22, 24–30, 35, 37, 39, 44, 45, 49, 52, 54], RANKL [25–27, 35, 38, 39, 43–46], pro-inflammatory cytokines [19, 22, 24, 29, 32, 34, 47, 48, 50, 54], as well as the inhibition and control of osteoclasts [19, 21,26,27, 30–32, 34–41, 43–44, 46–49, 52, 54], M1-type macrophages [29, 32, 34, 45], and Th17 [35]. These molecules and cells participate in bone resorption [1–6, 9] and their control or inhibition improves the therapy for bone resorption [1–6, 9]. In addition, the EVs increased levels of OPG [41–44], regulatory T cell [35], IL-10 [19, 29, 32, 47], and M2-type macrophage [18, 26, 32, 34, 45], collaborating with control bone resorption [3, 5–7].

In addition to these aspects, the EVs still promoted bone regeneration which enhances the quality and the quantity of bone [17, 18, 20, 21, 23–25, 27–31, 34–37, 39–41, 43, 49–54]. This characteristic collaborates with the balance of bone loss generated by different diseases linked to bone resorption.

Although the included studies are heterogeneous in terms of origin cells, doses, administration routes, and disease models, we addressed this variability by grouping the studies according to the disease investigated, as shown in Tables 2, 3, and 4. This approach allowed the identification of patterns that appear to be more consistent within each specific disease context. As a result, it can be observed that 14 [20, 25, 27, 28, 30, 35, 42, 44, 46–49, 52–54] of the 18 studies related to osteoporosis prioritized administration by intravenous route, with the effect being dose-dependent when different doses were compared within the same study [20, 44]. Regarding periodontitis, all studies applied EVs directly to the site of the bone defect resulting from the resorptive process [17–19, 21–24, 26, 31, 34, 37, 38, 45, 50]; due to the similarity of the outcomes, it is believed that in this case, lower doses may be more appropriate. It should be emphasized that these interpretations represent preliminary inferences, serving as initial indications rather than definitive conclusions. Future studies are needed to validate and strengthen these disease-specific observations.

Concerning the origin cell, new approaches are needed to determine the ideal one. Since the majority of studies demonstrated positive results for the control of bone resorption, considering accessibility, ease of obtaining, and ethical aspects is essential. Thus, bone marrow stem cells [19, 41, 46, 53] and macrophages [17, 21, 25, 37] can be prioritized in future studies.

A limitation of the present review is linked to the impossibility of the performance of meta-analysis. The meta-analysis was not conducted due to insufficient data. The necessary data for this analysis were published through graphs and images in the included studies in the present review. Thus, the precise collection of the means and standard deviations to control and treated groups to carry out the meta-analysis was impossible. The authors of published studies were contacted at three different times by email, however only three decided to furnish the requested information.

Another limitation was related to the risk of bias, as in several domains the risk was classified as unclear due to the lack of information regarding whether the assessed procedures were performed. This highlights the need for caution when extrapolating these findings to clinical trials and reinforces the necessity of additional studies before such translation can be considered. Additionally, the heterogeneity of origin cells, posology and experimental models represented another limitation, and as previously discussed, future studies focusing on addressing these aspects are required to enhance translational potential and support the development of future clinical applications.

Furthermore, considering the eligibility criteria, 24 articles were excluded after full-text reading, as shown in Table 1. The eligibility criteria addressed aspects included in the objectives of this systematic review. Therefore, in the absence of any essential data required to answer the proposed questions, the study was excluded and the reason for exclusion was justified.

Differences can be observed between animal and human studies, such as the use of certain vehicles like PBS [18–21, 23, 25–27, 34, 35, 37, 41–43, 45, 49, 54], the employment of simulated models to induce disease in animals [17–54], and some types of analysis [17–54]. Moreover, it is important to emphasize that early studies in animals are essential for testing initial hypotheses and assessing potential therapeutic effects prior to use in humans. Therefore, the EV-based therapy under investigation appears comparable across animal and human models, supporting a cautious but meaningful interpretation of the preclinical findings [57].

Despite these limitations, considering the descriptive data in this review, associated with the number of animals and statistical analysis of individual articles, as well as overall scientific quality of evidence, it is possible to infer the potential use of EVs for treating bone resorption-related diseases. Early-phase or Phase I/II translational studies that first assess safety, dosing, and feasibility in humans must be conducted to confirm the results obtained in this systematic review.

## References

[1] Teitelbaum SL (2000) Bone resorption by osteoclasts. Science 289(5484):1504–1508 10968780 10.1126/science.289.5484.1504

[2] Teitelbaum SL (2007) Osteoclasts: what do they do and how do they do it? Am J Pathol 170(2):427–435 17255310 10.2353/ajpath.2007.060834 PMC1851862

[3] Singh M, Singh P, Singh B, Sharma K, Kumar N, Singh D et al (2024) Molecular signaling pathways and microRNAs in bone remodeling: a narrative review. Dis. 10.3390/diseases12100252 10.3390/diseases12100252 PMC11507001 39452495

[4] Rumpler M, Wurger T, Roschger P, Zwettler E, Sturmlechner I, Altmann, et al (2013) Osteoclasts on bone and dentin in vitro: mechanism of trail formation and comparison of resorption behavior. Calcif Tissue Int 93(6):526–539 24022329 10.1007/s00223-013-9786-7 PMC3827903

[5] Wang Z, McCauley LK (2011) Osteoclasts and odontoclasts: signaling pathways to development and disease. Oral Dis 17(2):129–142 20659257 10.1111/j.1601-0825.2010.01718.x

[6] Iglesias-Linares A, Jr Hartsfield JK (2017) Cellular and molecular pathways leading to external root resorption. J Dent Res 96(2):145–152 27811065 10.1177/0022034516677539 PMC5331617

[7] Novak ML, Koh TJ (2013) Phenotypic transitions of macrophages orchestrate tissue repair. Am J Pathol 183(5):1352–1363 24091222 10.1016/j.ajpath.2013.06.034 PMC3969506

[8] Pillai NR, Aggarwal A, Orchard P (2022) Phenotype-autosomal recessive osteopetrosis. Bone 165:116577 36195244 10.1016/j.bone.2022.116577

[9] Hu Y, Wang Y, Chen T, Hao Z, Cai L, Li J (2021) Exosome: function and application in inflammatory bone diseases. Oxid Med Cell Longev 2021:6324912 34504641 10.1155/2021/6324912 PMC8423581

[10] Song H, Li X, Zhao Z, Qian J, Wang Y, Cui J et al (2019) Reversal of osteoporotic activity by endothelial cell-secreted bone targeting and biocompatible exosomes. Nano Lett 19(5):3040–3048 30968694 10.1021/acs.nanolett.9b00287

[11] Cui Y, Guo Y, Kong L, Shi J, Liu P, Li R et al (2021) A bone- targeted engineered exosome platform delivering siRNA to treat osteoporosis. Bioact Mater 10:207–221 34901540 10.1016/j.bioactmat.2021.09.015 PMC8636739

[12] Subarajan P, Arceo-Mendoza RM, Camacho PM (2024) Postmenopausal osteoporosis: a review of latest guidelines. Endocrinol Metab Clin North Am. 10.1016/j.ecl.2024.08.008 39448132 10.1016/j.ecl.2024.08.008

[13] Lee KS, Lee J, Kim HK, Yeom SH, Woo CH, Jung YJ et al (2021) Extracellular vesicles from adipose tissue-derived stem cells alleviate osteoporosis through osteoprotegerin and miR-21-5p. J Extracell Vesicles 10(12):e12152 34596354 10.1002/jev2.12152 PMC8485335

[14] Uenaka M, Yamashita E, Kikuta J, Morimoto A, Ao T, Mizuno H et al (2022) Osteoblast-derived vesicles induce a switch from bone-formation to bone-resorption in vivo. Nat Commun 13(1):1066 35210428 10.1038/s41467-022-28673-2 PMC8873258

[15] Page MJ, McKenzie JE, Bossuyt PM, Boutron I, Hoffmann TC, Mulrow CD et al (2021) The PRISMA 2020 statement: an updated guideline for reporting systematic reviews. BMJ. 10.1136/bmj.n71 33782057 10.1136/bmj.n71 PMC8005924

[16] Landis JR, Koch GG (1977) The measurement of observer agreement for categorical data. Biometrics 33(1):159–174 843571

[17] Cui Y, Hong S, Jiang W, Mao L, Lin K, Wang X (2025) Mitophagy-enhanced multimodal macrophage-derived hybrid nanovesicles for alleviating periodontitis-ulcerative colitis comorbidities: killing two birds with one stone. Chem Eng J 519:164678. 10.1016/j.cej.2025.164678

[18] Fan L, Wang T, Liu Y, Ma M, Liu G, Wang Y, Xia J, Gu Z, Hao Z (2025) Natural polyphenol-functionalized Schwann cell-derived exosomes as a temporal neuromodulation strategy for diabetic periodontitis therapy. ACS Nano 19(36):32482–32498 40908799 10.1021/acsnano.5c08885

[19] Guo Q, Ren S, Libonti A, Li J, Wang Z, Ren J, Zhang G, Gao L, Ba H, Shen Y, Li C (2025) Antler stem cell-derived exosomes restore periodontal homeostasis in a rat model with diabetic periodontitis through enhancing ROS scavenging and osteogenesis. Cell Death Discov 11:500. 10.1038/s41420-025-02800-6 41184223 10.1038/s41420-025-02800-6 PMC12583464

[20] Kim KY, Tsolmon KE, Bavuu Z, Noh CH, Kim HS, Jeong HS, Park D, Hong SC, Kim YB (2025) Osteoporosis-improving effects of extracellular vesicles from human amniotic membrane stem cells in ovariectomized rats. Int J Mol Sci 28(19):9503. 10.3390/ijms26199503 10.3390/ijms26199503 PMC12525326 41096767

[21] Lai S, Tang N, Guo J, Deng L, Yuan L, Zeng L, Yang L, Mu Y (2025) Immunomodulatory peptide DP7-C mediates macrophage-derived exosomal miR-21b to promote bone regeneration via the SOCS1/JAK2/STAT3 axis. Colloids Surf, B 253:114709. 10.1016/j.colsurfb.2025.114709 10.1016/j.colsurfb.2025.114709 40286607

[22] Li K, Gu X, Zhu Y, Guan N, Wang J, Wang L (2025) Human umbilical cord mesenchymal stem cells-derived exosomes attenuates experimental periodontitis in mice partly by delivering miRNAs. Int J Nanomedicine 20:2879–2899. 10.2147/IJN.S502192 40078652 10.2147/IJN.S502192 PMC11900796

[23] Liu S, Wang Z, Li Y, Pan Z, Huang L, Cui J, Zhang X, Yang M, Zhang Y, Li D, Sun H (2025) Erythropoietin-stimulated macrophage-derived extracellular vesicles in chitosan hydrogel rescue BMSCs fate by targeting EGFR to alleviate inflammatory bone loss in periodontitis. Adv Sci 12(23):e2500554. 10.1002/advs.202500554 10.1002/advs.202500554 PMC12199399 40289904

[24] Rojas C, García M, González-Osuna L, Campos-Mora M, de León EP, Sierra-Cristancho A, Terraza C, Cortez C, Sansores-España LD, Carvajal P, Bazoer J, Peng Q, Lawson C, Smyth LA, Pino-Lagos K, Vernal R (2025) Induced Treg-derived extracellular vesicles suppress CD4^+^ T-cell-mediated inflammation and ameliorate bone loss during periodontitis partly through CD73/adenosine-dependent immunomodulatory mechanisms. J Extracell Vesicles 14(7):e70118. 10.1002/jev2.70118 40620009 10.1002/jev2.70118 PMC12230360

[25] Chang W, Tian B, Qin Q, Li D, Zhang Y, Zhou C, Wu B, Zhang M, Shan H, Ni Y, Dong Q, Wang C, Zhou XZ, Bai J (2024) Receptor activator of nuclear factor Kappa-B-expressing mesenchymal stem cells-derived extracellular vesicles for osteoporosis therapy. ACS Nano 31(52):35368–35382. 10.1021/acsnano.4c12064 10.1021/acsnano.4c12064 39692894

[26] Huang Y, Li M, Liu Q, Song L, Wang Q, Ding P, Tian W, Guo S (2024) Small extracellular vesicles derived from lipopolysaccharide-preconditioned dental follicle cells inhibit cell apoptosis and alveolar bone loss in periodontitis. Arch Oral Biol. 10.1016/j.archoralbio.2024.105964 38582010 10.1016/j.archoralbio.2024.105964

[27] Wang ZX, Lin X, Cao J, Liu YW, Luo ZW, Rao SS, Wang Q, Wang YY, Chen CY, Zhu GQ, Li FX, Tan YJ, Hu Y, Yin H, Li YY, He ZH, Liu ZZ, Yuan LQ, Zhou Y, Wang ZG, Xie H (2024) Young osteocyte-derived extracellular vesicles facilitate osteogenesis by transferring tropomyosin-1. J Nanobiotechnology. 2 10.1186/s12951-024-02367-x. 10.1186/s12951-024-02367-x PMC11046877 38664789

[28] Xing Z, Guo L, Li S, Huang W, Su J, Chen X, Li Y, Zhang J. Skeletal muscle-derived exosomes prevent osteoporosis by promoting osteogenesis. Life Sci. 2024 Nov 15;357:123079. 10.1016/j.lfs.2024.123079. Epub 2024 Sep 24. PMID: 39326580. 10.1016/j.lfs.2024.123079 39326580

[29] Yuan N, Zhang W, Yang W, Ji W, Li J (2024) Exosomes derived from M2 macrophages prevent steroid-induced osteonecrosis of the femoral head by modulating inflammation, promoting bone formation and inhibiting bone resorption. J Orthop Surg Res. 10.1186/s13018-024-04711-1 38622659 10.1186/s13018-024-04711-1 PMC11020342

[30] Huang H, Ma S, Xing X, Su X, Xu X, Tang Q, Gao X, Yang J, Li M, Liang C, Wu Y, Liao L, Tian W (2023) Muscle-derived extracellular vesicles improve disuse-induced osteoporosis by rebalancing bone formation and bone resorption. Acta Biomater. 10.1016/j.actbio.2022.12.019 36526242 10.1016/j.actbio.2022.12.019

[31] Lu J, Yu N, Liu Q, Xie Y, Zhen L (2023) Periodontal ligament stem cell exosomes key to regulate periodontal regeneration by miR-31-5p in mice model. Int J Nanomedicine. 10.2147/IJN.S409664 37746047 10.2147/IJN.S409664 PMC10516219

[32] Luo H, Chen D, Li R, Li R, Teng Y, Cao Y, Zou X, Wang W, Zhou C (2023) Genetically engineered CXCR4-modified exosomes for delivery of miR-126 mimics to macrophages alleviate periodontitis. J Nanobiotechnology. 10.1186/s12951-023-01863-w 36991451 10.1186/s12951-023-01863-w PMC10061745

[33] Pan L, Zhang C, Zhang H, Ke T, Bian M, Yang Y, Chen L, Tan J (2023) Osteoclast-derived exosomal miR-5134-5p interferes with alveolar bone homeostasis by targeting the JAK2/STAT3 axis. Int J Nanomedicine. 10.2147/IJN.S413692 37441084 10.2147/IJN.S413692 PMC10335290

[34] Qiao X, Tang J, Dou L, Yang S, Sun Y, Mao H, Yang D (2023) Dental pulp stem cell-derived exosomes regulate anti-inflammatory and osteogenesis in periodontal ligament stem cells and promote the repair of experimental periodontitis in rats. Int J Nanomedicine. 10.2147/IJN.S420967 37608819 10.2147/IJN.S420967 PMC10441659

[35] Yang Z, Yang Z, Ding L, Liu C, Zhao F, Chen X, Du C (2023) Nanoengineering multifunctional extracellular vesicles availably mitigate bone loss in osteoporosis through binding to RANKL and rebalancing the Treg/Th17 cells. Chem Eng J. 10.1016/j.cej.2023.14339137484163

[36] Yang JG, Sun B, Wang Z, Li X, Gao JH, Qian JJ, Li J, Wei WJ, Zhang P, Wang W (2023) Exosome-targeted delivery of METTL14 regulates NFATc1 m6A methylation levels to correct osteoclast-induced bone resorption. Cell Death Dis. 10.1038/s41419-023-06263-4 37957146 10.1038/s41419-023-06263-4 PMC10643436

[37] Chen X, Wan Z, Yang L, Song S, Fu Z, Tang K, Chen L, Song Y (2022) Exosomes derived from reparative M2-like macrophages prevent bone loss in murine periodontitis models via IL-10 mRNA. J Nanobiotechnology. 10.1186/s12951-022-01314-y 35248085 10.1186/s12951-022-01314-y PMC8898524

[38] Shimizu Y, Takeda-Kawaguchi T, Kuroda I, Hotta Y, Kawasaki H, Hariyama T, Shibata T, Akao Y, Kunisada T, Tatsumi J, Tezuka KI (2022) Exosomes from dental pulp cells attenuate bone loss in mouse experimental periodontitis. J Periodontal Res. 10.1111/jre.12949 34826339 10.1111/jre.12949

[39] Wang R, Xu B (2022) TGFβ1-modified MSC-derived exosome attenuates osteoarthritis by inhibiting PDGF-BB secretion and H-type vessel activity in the subchondral bone. Acta Histochem. 10.1016/j.acthis.2022.151933 35933783 10.1016/j.acthis.2022.151933

[40] Wang Y, Zhou X, Wang D (2022) Mesenchymal stem cell-derived extracellular vesicles inhibit osteoporosis via microRNA-27a-induced inhibition of DKK2-mediated Wnt/β-catenin pathway. Inflammation. 10.1007/s10753-021-01583-z 34676493 10.1007/s10753-021-01583-z

[41] Yang Z, Liu X, Zhao F, Yao M, Lin Z, Yang Z, Liu C, Liu Y, Chen X, Du C (2022) Bioactive glass nanoparticles inhibit osteoclast differentiation and osteoporotic bone loss by activating lncRNA NRON expression in the extracellular vesicles derived from bone marrow mesenchymal stem cells. Biomaterials. 10.1016/j.biomaterials.2022.121438 35220020 10.1016/j.biomaterials.2022.121438

[42] Cui Y, Guo Y, Kong L, Shi J, Liu P, Li R, Geng Y, Gao W, Zhang Z, Fu D (2021) A bone-targeted engineered exosome platform delivering siRNA to treat osteoporosis. Bioact Mater. 10.1016/j.bioactmat.2021.09.015 34901540 10.1016/j.bioactmat.2021.09.015 PMC8636739

[43] Guo S, Gu J, Ma J, Xu R, Wu Q, Meng L, Liu H, Li L, Xu Y (2021) GATA4-driven miR-206-3p signatures control orofacial bone development by regulating osteogenic and osteoclastic activity. Theranostics. 10.7150/thno.58052 34373748 10.7150/thno.58052 PMC8344011

[44] Lee KS, Lee J, Kim HK, Yeom SH, Woo CH, Jung YJ, Yun YE, Park SY, Han J, Kim E, Sul JH, Jung JM, Park JH, Choi JS, Cho YW, Jo DG (2021) Extracellular vesicles from adipose tissue-derived stem cells alleviate osteoporosis through osteoprotegerin and miR-21-5p. J Extracell Vesicles. 10.1002/jev2.12152 34596354 10.1002/jev2.12152 PMC8485335

[45] Nakao Y, Fukuda T, Zhang Q, Sanui T, Shinjo T, Kou X, Chen C, Liu D, Watanabe Y, Hayashi C, Yamato H, Yotsumoto K, Tanaka U, Taketomi T, Uchiumi T, Le AD, Shi S, Nishimura F (2020) Exosomes from TNF-α-treated human gingiva-derived MSCs enhance M2 macrophage polarization and inhibit periodontal bone loss. Acta Biomater. 10.1016/j.actbio.2020.12.046 33359765 10.1016/j.actbio.2020.12.046 PMC7897289

[46] Xiao F, Zuo B, Tao B, Wang C, Li Y, Peng J, Shen C, Cui Y, Zhu J, Chen X (2021) Exosomes derived from cyclic mechanical stretch-exposed bone marrow mesenchymal stem cells inhibit RANKL-induced osteoclastogenesis through the NF-κB signaling pathway. Ann Transl Med. 10.21037/atm-21-1838 34268411 10.21037/atm-21-1838 PMC8246225

[47] Zhang L, Wang Q, Su H, Cheng J (2021) Exosomes from adipose derived mesenchymal stem cells alleviate diabetic osteoporosis in rats through suppressing NLRP3 inflammasome activation in osteoclasts. J Biosci Bioeng. 10.1016/j.jbiosc.2021.02.007 33849774 10.1016/j.jbiosc.2021.02.007

[48] Zhang L, Wang Q, Su H, Cheng J (2021) Exosomes from adipose tissues derived mesenchymal stem cells overexpressing MicroRNA-146a alleviate diabetic osteoporosis in rats. Cell Mol Bioeng. 10.1007/s12195-021-00699-4 35096186 10.1007/s12195-021-00699-4 PMC8761186

[49] Hu Y, Zhang Y, Ni CY, Chen CY, Rao SS, Yin H, Huang J, Tan YJ, Wang ZX, Cao J, Liu ZZ, Xie PL, Wu B, Luo J, Xie H (2020) Human umbilical cord mesenchymal stromal cells-derived extracellular vesicles exert potent bone protective effects by CLEC11A-mediated regulation of bone metabolism. Theranostics. 10.7150/thno.39238 32089743 10.7150/thno.39238 PMC7019162

[50] Wei J, Song Y, Du Z, Yu F, Zhang Y, Jiang N, Ge X (2020) Exosomes derived from human exfoliated deciduous teeth ameliorate adult bone loss in mice through promoting osteogenesis. J Mol Histol. 10.1007/s10735-020-09896-3 32656578 10.1007/s10735-020-09896-3

[51] Zhang X, You JM, Dong XJ, Wu Y (2020) Administration of mircoRNA-135b-reinforced exosomes derived from MSCs ameliorates glucocorticoid-induced osteonecrosis of femoral head (ONFH) in rats. J Cell Mol Med. 10.1111/jcmm.16006 33089961 10.1111/jcmm.16006 PMC7754047

[52] Chen CY, Rao SS, Tan YJ, Luo MJ, Hu XK, Yin H, Huang J, Hu Y, Luo ZW, Liu ZZ, Wang ZX, Cao J, Liu YW, Li HM, Chen Y, Du W, Liu JH, Zhang Y, Chen TH, Liu HM, Wu B, Yue T, Wang YY, Xia K, Lei PF, Tang SY, Xie H (2019) Extracellular vesicles from human urine-derived stem cells prevent osteoporosis by transferring CTHRC1 and OPG. Bone Res. 10.1038/s41413-019-0056-9 31263627 10.1038/s41413-019-0056-9 PMC6594995

[53] Luo ZW, Li FX, Liu YW, Rao SS, Yin H, Huang J, Chen CY, Hu Y, Zhang Y, Tan YJ, Yuan LQ, Chen TH, Liu HM, Cao J, Liu ZZ, Wang ZX, Xie H (2019) Aptamer-functionalized exosomes from bone marrow stromal cells target bone to promote bone regeneration. Nanoscale. 10.1039/c9nr02791b 31660556 10.1039/c9nr02791b

[54] Song H, Li X, Zhao Z, Qian J, Wang Y, Cui J, Weng W, Cao L, Chen X, Hu Y, Su J (2019) Reversal of osteoporotic activity by endothelial cell-secreted bone targeting and biocompatible exosomes. Nano Lett. 10.1021/acs.nanolett.9b00287 30968694 10.1021/acs.nanolett.9b00287

[55] Xu R, Shen X, Si Y, Fu Y, Zhu W, Xiao T, Fu Z, Zhang P, Cheng J, Jiang H (2018) MicroRNA-31a-5p from aging BMSCs links bone formation and resorption in the aged bone marrow microenvironment. Aging Cell. 10.1111/acel.12794 29896785 10.1111/acel.12794 PMC6052401

[56] Xie Z, Shen Z, Zhan P et al (2021) Functional dental pulp regeneration: basic research and clinical translation. Int J Mol Sci 22:8991 34445703 10.3390/ijms22168991 PMC8396610

[57] Lener T, Gimona M, Aigner L, Börger V, Buzas E, Camussi G, Chaput N, Chatterjee D, Court FA, Del Portillo HA, O’Driscoll L, Fais S, Falcon-Perez JM, Felderhoff-Mueser U, Fraile L, Gho YS, Görgens A, Gupta RC, Hendrix A, Hermann DM, Hill AF, Hochberg F, Horn PA, de Kleijn D, Kordelas L, Kramer BW, Krämer-Albers EM, Laner-Plamberger S, Laitinen S, Leonardi T, Lorenowicz MJ, Lim SK, Lötvall J, Maguire CA, Marcilla A, Nazarenko I, Ochiya T, Patel T, Pedersen S, Pocsfalvi G, Pluchino S, Quesenberry P, Reischl IG, Rivera FJ, Sanzenbacher R, Schallmoser K, Slaper-Cortenbach I, Strunk D, Tonn T, Vader P, van Balkom BW, Wauben M, Andaloussi SE, Théry C, Rohde E, Giebel B (2015) Applying extracellular vesicles based therapeutics in clinical trials - an ISEV position paper. J Extracell Vesicles 4:30087. 10.3402/jev.v4.30087 26725829 10.3402/jev.v4.30087 PMC4698466

[58] Pi Z, Wu Y, Wang X, Li P, Wang R (2025) Exosomal Manf originated from endothelium regulated osteoclast differentiation by down-regulating NF-κB signaling pathway. J Orthop Surg Res 7(1):349. 10.1186/s13018-025-05671-w 10.1186/s13018-025-05671-w PMC11978012 40197525

[59] Li F, Zhao X, Zhang Y, Zhuang Q, Wang S, Fang X et al (2024) Exosomal circFAM63Bsuppresses bone regeneration of postmenopausal osteoporosis via regulating miR-578/HMGA2 axis. J Orthop Res. 10.1002/jor.25776 38151824 10.1002/jor.25776

[60] Zhou Y, Hu G (2024) M2 macrophages-derived exosomes regulate osteoclast differentiation by the CSF2/TNF-α axis. BMC Oral Health. 10.1186/s12903-023-03842-x 38238696 10.1186/s12903-023-03842-x PMC10795354

[61] Cai M, Peng H, Liu M, Huang M, Zheng W, Zhang G et al (2023) Vascular pericyte-derived exosomes inhibit bone resorption via Traf3. Int J Nanomedicine. 10.2147/IJN.S438229 38046234 10.2147/IJN.S438229 PMC10693246

[62] Choi JH, Sung SE, Kang KK, Lee S, Sung M, Park WT, Kim YI, Seo MS, Lee GW (2024) Extracellular vesicles from human adipose tissue-derived mesenchymal stem cells suppress RANKL-induced osteoclast differentiation via miR122-5p. Biochem Genet. 10.1007/s10528-023-10569-5 38017286 10.1007/s10528-023-10569-5

[63] Duan J, Li H, Wang C, Yao J, Jin Y, Zhao J, Zhang Y, Liu M, Sun H (2023) BMSC-derived extracellular vesicles promoted osteogenesis via Axin2 inhibition by delivering MiR-16-5p. Int Immunopharmacol. 10.1016/j.intimp.2023.110319 37216799 10.1016/j.intimp.2023.110319

[64] Li X, Jiang Y, Liu X, Fu J, Du J, Luo Z, Xu J, Bhawal UK, Liu Y, Guo L (2023) Mesenchymal stem cell-derived apoptotic bodies alleviate alveolar bone destruction by regulating osteoclast differentiation and function. Int J Oral Sci. 10.1038/s41368-023-00255-y 38040672 10.1038/s41368-023-00255-y PMC10692139

[65] Li S, Wu C, Lin S, Wen Z, Luo W, Li C, Wang X, Li X, Gao L, Ding Y (2023) HUCMSC-derived Exosomes Suppress the Titanium Particles-induced Osteolysis in Mice through Inhibiting CCL2 and CCL3. Orthop Surg. 10.1111/os.13608 36720704 10.1111/os.13608 PMC9977603

[66] Pan B, Zhang Z, Wu X, Xian G, Hu X, Gu M, Zheng L, Li X, Long L, Chen W, Sheng P (2023) Macrophage-derived exosomes modulate wear particle-induced osteolysis via mir-3470b targeting TAB3/NF-κB signaling. Bioact Mater. 10.1016/j.bioactmat.2023.02.028 36911207 10.1016/j.bioactmat.2023.02.028 PMC9999169

[67] Sedik AS, Kawana KY, Koura AS, Mehanna RA (2023) Biological effect of bone marrow mesenchymal stem cell- derived extracellular vesicles on the structure of alveolar bone in rats with glucocorticoid-induced osteoporosis. BMC Musculoskelet Disord. 10.1186/s12891-023-06276-2 36932362 10.1186/s12891-023-06276-2 PMC10022145

[68] Xie J, Hu Y, Li H, Wang Y, Fan X, Lu W, Liao R, Wang H, Cheng Y, Yang Y, Wang J, Liang S, Ma T, Su W (2023) Targeted therapy for peri-prosthetic osteolysis using macrophage membrane-encapsulated human urine-derived stem cell extracellular vesicles. Acta Biomater. 10.1016/j.actbio.2023.02.003 36773884 10.1016/j.actbio.2023.02.003

[69] Yin S, Lin S, Xu J, Yang G, Chen H, Jiang X (2023) Dominoes with interlocking consequences triggered by zinc: involvement of microelement-stimulated MSC-derived exosomes in senile osteogenesis and osteoclast dialogue. J Nanobiotechnology. 10.1186/s12951-023-02085-w 37741978 10.1186/s12951-023-02085-w PMC10518091

[70] Zhang B, Lai RC, Sim WK, Lim SK (2023) Therapeutic efficacy of mesenchymal stem/stromal cell small extracellular vesicles in alleviating arthritic progression by restoring macrophage balance. Biomolecules 13(10):1501. 10.3390/biom13101501 37892183 10.3390/biom13101501 PMC10605110

[71] Ren L, Zeng F, Deng J, Bai Y, Chen K, Chen L, Sun L (2022) Inflammatory osteoclasts-derived exosomes promote bone formation by selectively transferring lncRNA LIOCE into osteoblasts to interact with and stabilize Osterix. FASEB J. 10.1096/fj.202101106RR 35032415 10.1096/fj.202101106RR

[72] Ye Q, Xu H, Liu S, Li Z, Zhou J, Ding F, Zhang X, Wang Y, Jin Y, Wang Q (2022) Apoptotic extracellular vesicles alleviate Pg-LPS induced inflammatory responses of macrophages via AMPK/SIRT1/NF-κB pathway and inhibit osteoclast formation. J Periodontol. 10.1002/JPER.21-0657 35499816 10.1002/JPER.21-0657

[73] Gao XR, Ge J, Li WY, Zhou WC, Xu L, Geng DQ (2020) Mir-34a carried by adipocyte exosomes inhibits the polarization of M1 macrophages in mouse osteolysis model. J Biomed Mater Res A. 10.1002/jbm.a.37088 32803914 10.1002/jbm.a.37088

[74] Li H, Fan XL, Wang YN, Lu W, Wang H, Liao R, Zeng M, Yang JX, Hu Y, Xie J (2021) Extracellular vesicles from human urine-derived stem cells ameliorate particulate polyethylene-induced osteolysis. Int J Nanomedicine. 10.2147/IJN.S325646 34785895 10.2147/IJN.S325646 PMC8579861

[75] Wang Q, Shen X, Chen Y, Chen J, Li Y (2021) Osteoblasts-derived exosomes regulate osteoclast differentiation through miR-503-3p/Hpse axis. Acta Histochem. 10.1016/j.acthis.2021.151790 34592492 10.1016/j.acthis.2021.151790

[76] Yang G, Yu H, Liu Y, Ye W, Zhu G, Yan A, Tan Q, Mei H (2021) Serum-derived exosomes from neurofibromatosis type 1 congenital tibial pseudarthrosis impaired bone by promoting osteoclastogenesis and inhibiting osteogenesis. Exp Biol Med. 10.1177/1535370220962737 10.1177/1535370220962737 PMC7871115 33023333

[77] Hayashi C, Fukuda T, Kawakami K, Toyoda M, Nakao Y, Watanabe Y, Shinjo T, Sano T, Iwashita M, Yotsumoto K, Shida M, Taketomi T, Sanui T, Uchiumi T, Kanematsu T, Nishimura F (2022) MiR-1260b inhibits periodontal bone loss by targeting ATF6β mediated regulation of ER stress. Front Cell Dev Biol. 10.3389/fcell.2022.1061216 36531939 10.3389/fcell.2022.1061216 PMC9748617

[78] Qayoom I, Teotia AK, Kumar A (2020) Nanohydroxyapatite based ceramic carrier promotes bone formation in a femoral neck canal defect in osteoporotic rats. Biomacromol. 10.1021/acs.biomac.9b01327 10.1021/acs.biomac.9b01327 31637919

[79] Yun B, Maburutse BE, Kang M, Park MR, Park DJ, Kim Y, Oh S (2020) Short communication: dietary bovine milk-derived exosomes improve bone health in an osteoporosis-induced mouse model. J Dairy Sci. 10.3168/jds.2019-17501 32622594 10.3168/jds.2019-17501

[80] Cui Y, Fu S, Sun D, Xing J, Hou T, Wu X (2019) EPC-derived exosomes promote osteoclastogenesis through LncRNA-MALAT1. J Cell Mol Med. 10.1111/jcmm.14228 31025509 10.1111/jcmm.14228 PMC6533478

[81] Hu Y, Xu R, Chen CY, Rao SS, Xia K, Huang J, Yin H, Wang ZX, Cao J, Liu ZZ, Tan YJ, Luo J, Xie H (2019) Extracellular vesicles from human umbilical cord blood ameliorate bone loss in senile osteoporotic mice. Metabolism. 10.1016/j.metabol.2019.01.009 30668962 10.1016/j.metabol.2019.01.009

[82] Hooijmans CR, Rovers MM, de Vries RB, Leenaars M, Ritskes-Hoitinga M, Langendam MW (2014) SYRCLE’s risk of bias tool for animal studies. BMC Med Res Methodol 14:43 24667063 10.1186/1471-2288-14-43 PMC4230647

